# From expression footprints to causal pathways: contextualizing large signaling networks with CARNIVAL

**DOI:** 10.1038/s41540-019-0118-z

**Published:** 2019-11-11

**Authors:** Anika Liu, Panuwat Trairatphisan, Enio Gjerga, Athanasios Didangelos, Jonathan Barratt, Julio Saez-Rodriguez

**Affiliations:** 1 Heidelberg University, Faculty of Medicine, and Heidelberg University Hospital, Institute of Computational Biomedicine, Bioquant, 69120 Heidelberg, Germany; 2 RWTH Aachen University, Faculty of Medicine, Joint Research Centre for Computational Biomedicine (JRC-COMBINE), 52074 Aachen, Germany; 3 Department of Infection, Immunity and Inflammation, University of Leicester, Leicester, UK

**Keywords:** Regulatory networks, Software

## Abstract

While gene expression profiling is commonly used to gain an overview of cellular processes, the identification of upstream processes that drive expression changes remains a challenge. To address this issue, we introduce CARNIVAL, a causal network contextualization tool which derives network architectures from gene expression footprints. CARNIVAL (CAusal Reasoning pipeline for Network identification using Integer VALue programming) integrates different sources of prior knowledge including signed and directed protein–protein interactions, transcription factor targets, and pathway signatures. The use of prior knowledge in CARNIVAL enables capturing a broad set of upstream cellular processes and regulators, leading to a higher accuracy when benchmarked against related tools. Implementation as an integer linear programming (ILP) problem guarantees efficient computation. As a case study, we applied CARNIVAL to contextualize signaling networks from gene expression data in IgA nephropathy (IgAN), a condition that can lead to chronic kidney disease. CARNIVAL identified specific signaling pathways and associated mediators dysregulated in IgAN including Wnt and TGF-β, which we subsequently validated experimentally. These results demonstrated how CARNIVAL generates hypotheses on potential upstream alterations that propagate through signaling networks, providing insights into diseases.

## Network inference: causal reasoning from expression and prior knowledge

Changes in gene expression are generally indirect consequences of upstream dysregulation, and it is often important to understand what caused it. A team of researchers led by Julio Saez-Rodriguez from Heidelberg and Aachen has developed CARNIVAL to infer causal networks of proteins upstream of gene expression. In the first step, dysregulated transcription factors are inferred from gene expression. Subsequently, an algorithm finds explanations for their up- or down-regulation through known protein–protein interactions using an ILP formulation. Optionally, known targets of perturbation can be provided. This integration of different sources of prior knowledge led to higher performance than alternative methods and was able to identify key pathways and proteins, including TGFβ signaling and β-Catenin, in a case study on IgA nephropathy. Overall, CARNIVAL may provide clues to understand causal mechanisms in diseases and treatments.

## Introduction

Cells possess a sophisticated and finely tuned signaling architecture, and its dysregulation can alter cellular behavior leading to many diseases. A better understanding of signaling networks, therefore, allows us to gain insights into disease processes and to prioritize potential targets for drug development.

Signaling networks are context specific. A network for a specific context can be inferred from dedicated data computationally. This inference can be performed based on phosphoproteomics data that directly measure key signaling players such as receptors and kinases,^1,2^ preferably in combination with prior knowledge.^3^ However, the availability of phosphoproteomics data is often limited while gene expression data are more abundant. The inference of signaling networks based on gene expression is, therefore, an attractive approach to uncover the organization of cellular signal transduction.

There are multiple computational tools which allow the inference of regulatory signaling networks from gene expression data. Many of these methods assume gene expression levels as a proxy for signaling protein activities and use them to construct networks.^4^ For instance, Huang and Fraenkel mapped transcriptomics data onto signaling pathways and then applied a Steiner’s tree algorithm for network contextualization.^5^ Such methods can provide valuable insight, but are limited by the fact that the abundance and activities of signaling proteins only partially correlate with gene expression.^6^

To overcome this limitation, one can alternatively identify upstream signaling regulators from the profiles of downstream gene targets. One approach is to analyze gene expression footprints of signaling pathways obtained from perturbation experiments.^7–9^ Another one is to predict transcription factor (TF) activities based on their regulons.^10,11^ However, these approaches do not provide information on the topology of signaling pathways. This information can be obtained by applying network-based approaches that can incorporate the network structure as prior information.

Given a starting prior knowledge network (PKN), upstream regulators can be inferred from downstream signaling targets in the form of a sub-network that infers direct connections and further upstream signaling events, as implemented by Melas et al.^12–14^ These tools, however, only take the PKN as prior knowledge. The tool X2K, in contrast, uses expression footprint as prior knowledge to link gene expression to upstream regulatory kinases using TF and kinase enrichment, but without considering the causality of the cascades.^15^

**Fig. 1:**
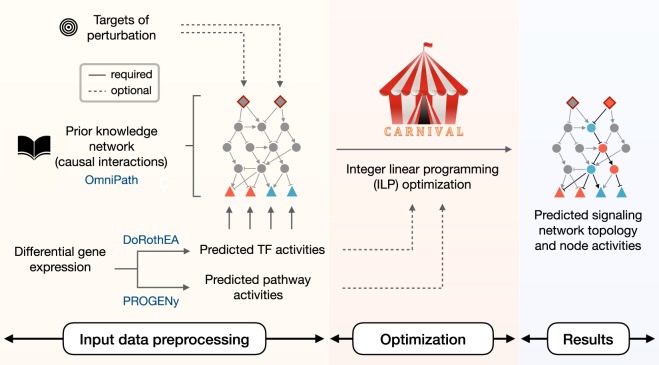
CARNIVAL pipeline. The CARNIVAL pipeline requires as input a prior knowledge network and differential gene expression. The information on perturbed targets and their effects can be assigned (Standard CARNIVAL “StdCARNIVAL”) or omitted (InvCARNIVAL). The differential gene expression is used to infer transcription factor (TF) activities with DoRothEA, which are subsequently discretized in order to formulate ILPconstraints. As a result, CARNIVAL derives a family of highest scoring networks which best explain theinferred TF activities. Continuous pathway and TF activities can be additionally considered in the objective function

We set out to integrate the causal network approach with expression footprints to infer the whole signaling cascade. For this, we developed the causal reasoning tool CARNIVAL (CAusal Reasoning pipeline for Network identification using Integer VALue programming). CARNIVAL expands an integer linear programming (ILP) implementation for causal reasoning^12^ to integrate information from TF and signaling pathway activity scoring. In addition, it can be applied not only to perturbation experiments, as in the original implementation^12^, but also generally to compare between two or more conditions. CARNIVAL uses a comprehensive collection of pathway resources available in OmniPath as PKN,^16^ though other sources can be used (Fig. 1). We performed a benchmarking study using the SBVimprover Species Translation Challenge dataset^17^ and compared its performance to an alternative causal reasoning network contextualization tool CausalR.^13^ As a case study, we apply CARNIVAL to glomerular gene expression data on IgA nephropathy (IgAN), a common chronic kidney disease (CKD) accounting for 35% of all renal transplantations in adults,^18^ in order to gain insights on the cellular processes that regulate its pathophysiology. These were confirmed by independent experimental validation.

## Results

### Benchmarking on the SBV improver dataset

To evaluate the performance of the CARNIVAL pipeline, we applied it to the SBVimprover Species Translation Challenge dataset which provides phosphorylation and gene expression data for multiple perturbations.^17^ We applied both the Standard CARNIVAL “StdCARNIVAL” and Inverse CARNIVAL “InvCARNIVAL” pipelines to evaluate the effect of information from the perturbation targets onto the resulting networks. The results from both pipelines were then compared to the ones generated by CausalR as well as GSEA.

**Fig. 2:**
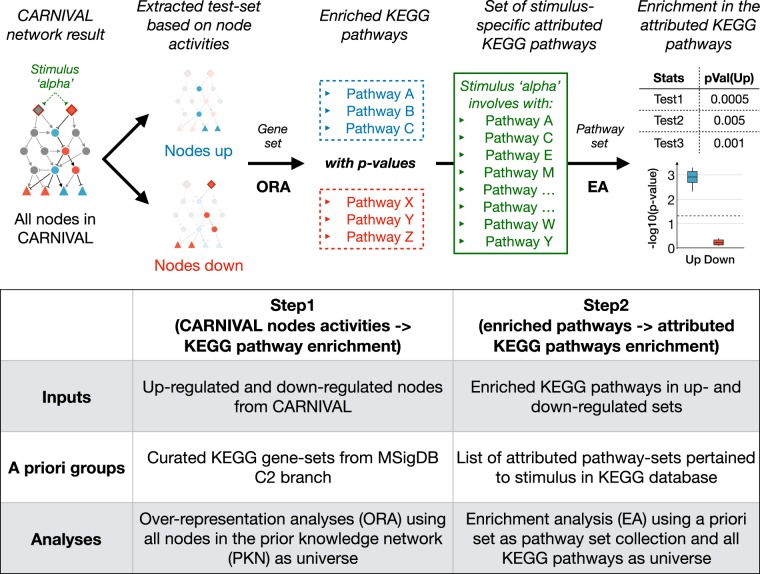
Two-step inference analysis to determine whether relevant molecular processes were identified in CARNIVAL. First, dysregulated pathways were inferred by over-representation of the nodes in CARNIVAL solution networks based on the KEGG pathway sets in MSigDB. In the second step, an enrichment analysis was performed on the identified dysregulated pathways using stimulus specific pathways as prior set. The distributions of *p*-values from multiple statistical tests are reported as final result. A significant enrichment of the attributed pathways in the direction that the target protein is perturbed is expected

This study provides phosphoprotein data, which in principle lend itself to validate the estimated activity of nodes with CARNIVAL. However, only 3-to-4 out of 19 phosphosites in the phosphoprotein dataset could be mapped to CARNIVAL node activities per condition, which is insufficient for statistical analyses (see Methods). We therefore used an alternative validation. We first determined which pathways are known to be linked to the perturbations by different molecules according to the KEGG database;^19^ referred to perturbation-attributed pathways henceforth. We then performed an enrichment analysis to define whether they are more up- or down-regulated with a two-step inference approach (see Methods and Fig. 2). This gives insights into how well expected pathways and their regulatory direction are captured by CARNIVAL. As all 20 perturbations which were investigated have activatory effects, we expect significant enrichments in the up-regulated direction.

### Incorporation of predicted TF and pathway activities

The normalized enrichment scores (NESs) from DoRothEA were used as an estimate for the degree of dysregulation (see Methods). In comparison to the results from Melas et al.’s pipeline without the integration of TF weights, the results from StdCARNIVAL showed that significant enrichment of the perturbation-attributed pathway set in up-regulated pathways is only achieved for IL1-β (IL1B) and TGF-ɑ (TGFA) with the introduction of TF weights (Supplementary Fig. S1). In InvCARNIVAL, where the targets of perturbations are not known, the results with pathway weights from PROGENy showed a significant enrichment of perturbation-attributed pathways in up-regulated pathways for PDGF-β (PDGFB), IL1-β, EGF, TGF-ɑ, and flagellin while only TGF-ɑ and FSL1 were significant without pathway weights (Supplementary Fig. S2). TGF-ɑ was more enriched in activated than in inhibited pathways with pathway weights but this trend was inverse for IGF2 and AREG. While this implementation could not capture all expected changes, PROGENy weight still provides an overall improvement in detecting more dysregulated pathways in the up-regulated direction (5 versus 2). Given that improved performance was found with the implementation of TF and pathway weights, these were implemented in our subsequent benchmarking.

To illustrate the values of the CARNIVAL pipeline with respect to the ones of DoRothEA and PROGENy, we compared CARNIVAL enrichment results to the ones generated solely from either DoRothEA or PROGENy. Results show good agreement to the ones of StdCARNIVAL but it should be noted that positive results are not always confirmed by both methods (Supplementary Text S4).

### Comparison of StdCARNIVAL, InvCARNIVAL, GSEA, and CausalR

**Fig. 3:**
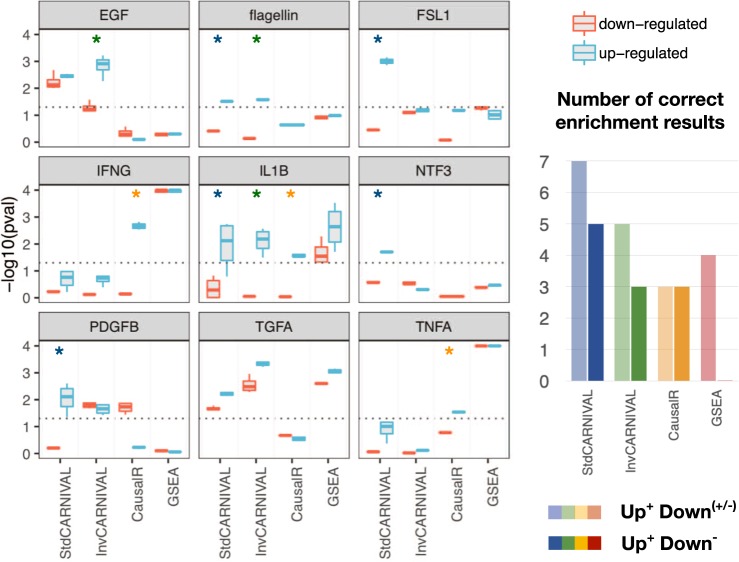
Comparison of the enrichment results of the perturbation-attributed pathway set in dysregulated pathways inferred with different tools. An enrichment of the perturbation-attributed pathway set among the significant pathways was determined. The significance level of 0.05 is indicated by the dotted lines. Asterisks (*) indicate the clear directionality of results where the enrichment results are significant in the up-regulated set and insignificant in the down-regulated set

To benchmark CARNIVAL results against related tools, we applied the two-step inference approach also on their results and made comparisons. As an overview, the perturbations of the significant up-regulated sets found in InvCARNIVAL (PDGF-β, IL1-β, EGF, TGF-ɑ, and flagellin) were also identified in StdCARNIVAL (Fig. 3; Supplementary Fig. S3). In contrast, NTF3 and FSL1 only showed a significant enrichment of the up-regulated gene sets in StdCARNIVAL. This suggests that a wider coverage of pathways can be detected by StdCARNIVAL, where the perturbation target is known. The same number of enrichment of activated pathways in the perturbation-attributed pathways (up-regulated) captured with InvCARNIVAL is slightly higher than the one with pathway inference from differential gene expression directly (*n* = 5 versus *n* = 4). TGF-ɑ and IL1-β were captured with both approaches, while IFN-γ (IFNG) and TNF-ɑ (TNFA) were only significantly enriched in activated pathways inferred by GSEA, and IL1-β, PDGF-β, and EGF by InvCARNIVAL. However, the trend of clear directionality, i.e. significant in the up-regulated gene sets and insignificant in the down-regulated gene sets, was only captured with InvCARNIVAL. CausalR captured a significant enrichment of activated pathways in the perturbation-attributed pathway set for IFN-γ, IL1-β, and TNF-ɑ, showing an equal performance to InvCARNIVAL in terms of detecting non-ambiguous directionality but still captured less numbers of up-regulated pathways than GSEA and Std/InvCARNIVAL (Fig. 3).

We then compared the network topology of the CARNIVAL versus CausalR networks. We found a bias towards hub nodes for CausalR but not for CARNIVAL. The degree distribution of edges in CARNIVAL was very similar to the one of the PKN, see Supplementary Fig. S4. This trend was not affected by different beta parameters in (0.03; 0.1; 0.3; 0.5; 0.8) (result not shown).

### Inferring signaling networks in IgAN

We applied CARNIVAL to identify the regulatory signaling network that governs the pathophysiological mechanism of IgAN. It is known from the literature that the pathogenic IgA-containing immune complexes trigger the activation of inflammation and fibrosis^20^ but the dysregulation at the molecular level is yet to be elucidated. Further improvement in early diagnosis and treatment of IgAN are still needed and can only be achieved by a better understanding of the disease’s mechanisms to design appropriate treatments.

### InvCARNIVAL results

**Fig. 4:**
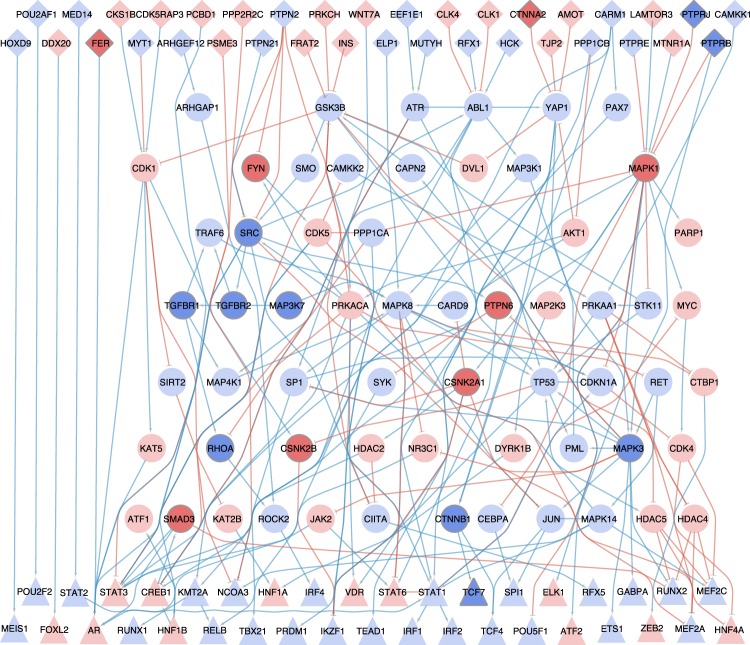
IgAN-contextualized network from CARNIVAL. The network summarizes the CARNIVAL results for node penalty *β* = 0.8. This network consists of 43 TFs, 37 input nodes and 62 associated nodes which are connected through 231 edges. Up-regulated nodes and activatory reactions are indicated in blue while down-regulated nodes and inhibitory edges are colored in red. Triangles correspond to transcription factors, diamonds represent input nodes and circlescor respond to purely inferred nodes. Members of the most dysregulated gene set, i.e. adherens junctions, are labeled by more intense background colors

In this study, we generated the causal networks from the differential gene expression in glomeruli between groups of healthy subjects versus IgAN patients. Given that the node penalty did not affect the performance but might result in minor fluctuations, this analysis was performed with different node penalties to achieve more robust results (β in (0.03; 0.1; 0.3; 0.5; 0.8), see Fig. 4 for β = 0.8 and Supplementary Fig. S5 for all betas). Given that the adherens junction set is the most dysregulated one, the set members are highlighted in the network. Thereby, only one transcription factor (TCF7) inferred by DoRothEA is represented in this gene set, while 13 associated nodes and four input nodes are solely inferred by causal reasoning with CARNIVAL. Hence, CARNIVAL was able to capture more pathway members and their connections than through TF-regulons alone.

In addition, we performed down-sampling and network randomization via re-shuffling of TF scores and labels to assess the robustness of CARNIVAL results for the IgAN datasets (see Supplementary Fig. S6). We observed that the inferred node activities of CARNIVAL networks from ±70% down-sampling datasets is relatively similar to the true IgAN network inferred from the full dataset (Jaccard similarity measure: 0.448 ± 0.051 [mean ± S.D.]). In contrast, a major difference was observed when compared the true IgAN network with the randomized networks from the re-shuffling dataset (Jaccard similarity measure: 0.065 ± 0.032 [mean ± S.D.]). These results suggest that CARNIVAL provides robust results significantly different from random networks (*p*-value < 0.001).

### Topological analyses and text-mining results of CARNIVAL networks

To identify the central regulatory nodes in the IgAN inferred networks, network’s properties of the results generated from different node penalties parameters were extracted. These measures include in-, out-, and all-degree of interactions as well as betweenness, hub-scor,e and authority-score of nodes in the networks (see Methods). The Top-20 results in each category are shown in Supplementary Table S3 and the robustness of results are shown in Supplementary Fig. S7. Of note, signaling molecules which are related to p53 pathway and cell cycle regulation including CDK1, ATM, and TP53 appeared frequently in the top list. In addition, the ERK/MAPK pathway represented by MAPK1 (ERK-2) and MAPK3 (ERK-1) was shown to have high-degree of connectivity where MAPK3 has a very high hub-score. The distributions of network topology measures for these nodes from the IgAN networks are statistically different comparing to the ones from randomized PKNs with the same degree distribution (average *p*-value = 0.0135). This suggests that central nodes in the IgAN network are not necessarily the existing hub nodes in the PKN. In addition, TP53 is the highest scoring node in randomized networks while MAPK1 and MAPK3 are often found to rank higher in the IgAN network. Note that a similar finding was observed for GSK-3β (GSK3B), highlighting the potential involvement of PI3K/Akt pathway (together with AKT1) as well as Wnt pathway in the molecular pathogenesis of IgAN.

Besides topological analyses, we systematically searched in the literature to identify whether the signaling proteins identified to be dysregulated in CARNIVAL have support for the roles in the pathophysiology of IgAN (see Methods and Supplementary Table S4). We observed that the molecules in the MAPK pathway and PI3K/Akt pathways, which are previously supported by topological analysis, also have multiple hits (7 hits for MAPK1/MAPK3 and 3 hits for AKT1). On the other hand, we identified many molecules which are in the top list of network topology measures but with no hit on text-mining results. These include down-regulated MAPK14 in the p38 MAPK pathway, down-regulated GSK3B in the PI3K/Akt and Wnt pathways, and up-regulated CDK5 in the cell cycle circuit. Also, we identified RhoA (RHOA) and β-Catenin (CTNNB1) as the components in the TGF-β and Wnt pathways having a few literature support with 2 and 3 hits, respectively. These less or not characterized proteins are candidate players in IgAN, pending validation.

### Inferred dysregulated cellular processes by CARNIVAL

**Fig. 5:**
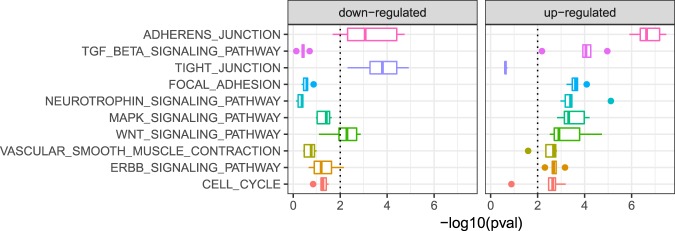
Dysregulated cellular processes in IgAN. Up- and down-regulated pathways are shown with decreasing median significance from top to bottom. The significance level is 0.01. Among others, these point to podocyte injury and the disruption of the slit diaphragms, as well as fibrosis

Dysregulated pathways were inferred by over-representation analysis of the CARNIVAL nodes in the KEGG gene sets. The most significantly up- and down-regulated pathways were identified by median *p*-value over different node penalties (Fig. 5). Among these, known drivers of renal fibrosis including TGF-β, Wnt, and EGFR/ErbB signaling stand out.^21–23^ The TGF-b and EGFR/ErbB pathways are significantly over-represented in CARNIVAL with clear directionality while Wnt and TGF-β pathways are ambiguous in GSEA (Supplementary Table S5). Additionally, focal adhesion was reported as an activated process in CARNIVAL and GSEA. Interactions between extracellular matrix and the cytoskeleton are particularly important in matrix-producing cells like fibroblasts and mesangial cells in the kidney.^24,25^ The enrichments of MAPK, Wnt, TGF-β, and cell cycle pathways were also supported by topological network analysis and text-mining results.

While CARNIVAL predicts an upregulation for Wnt and TGF-β (*p*-value: 0.0013 and 0.0014, respectively), GSEA analysis predicts these two pathways to be both up- and down-regulated in GSEA, hence being unclear in directionality (see details in Supplementary Table S5). These pathways are therefore worth to be investigated experimentally to confirm the validity of results from the two approaches.

### Fluorescence immunohistology detection of RhoA and β-catenin

**Fig. 6:**
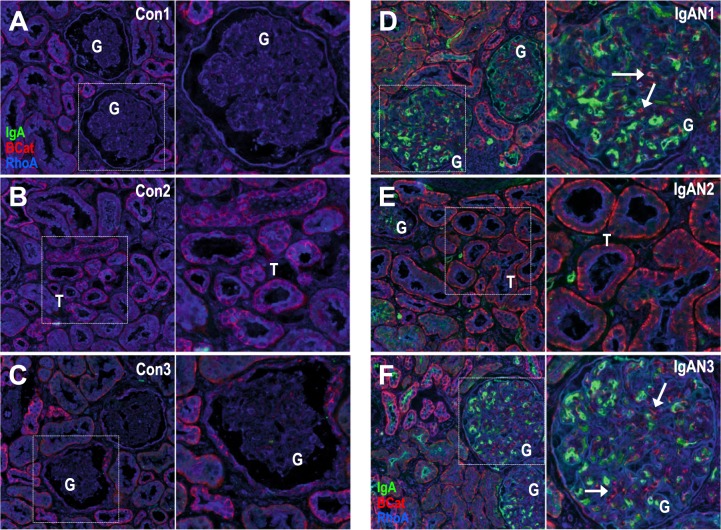
Validation experiment. IgA (green) beta-catenin (red) and RhoA (blue) staining was performed in human biopsies collected from either healthy pre-transplantation control donors(Con1-3; **a**–**c**) or diagnosed IgAN patients (IgAN1-3; **d**–**f**). 3 representative examples are shown and areas with glomeruli (G) and proximal tubules (T) are indicated. Accumulation of IgA (green) is the pathological hallmark of IgAN and there is no IgA staining in control specimens (**a**–**c**). RhoA immunostaining (blue) seems to be ubiquitous and dispersed in tubules and glomeruli. beta-catenin31 (red) is elevated in IgAN biopsies and there is an increase in beta-catenin cellular staining in glomeruli (arrows). Dotted white boxes depict highlighted areas magnified on left panels. All sections were 10 µm thick scanned with a 20x lens

The role of MAPK and PI3K/Akt signaling on IgAN’s pathophysiology have been described in previous studies.^26–29^ We therefore focused on validating key components of the TGF-β and Wnt signaling pathways with less literature support and where CARNIVAL and GSEA provided inconclusive results. We chose RhoA (RHOA) and β-catenin (CTNNB1) for fluorescence immunostaining on human renal biopsies from healthy pre-transplantation (controls) and biopsies from diagnosed IgAN patients (see Fig. 6 and Supplementary Text S5). Both RhoA and β-catenin genes were down-regulated comparing IgAN to control samples while the corresponding KEGG pathway enrichment scores showed a deregulation yet unclear direction. In contrast, CARNIVAL predicts that signaling activities are relatively increased for both when compared the ones from IgAN patients against healthy donors. Using immunohistology, mesangial IgA (green) was present in IgAN but absent in control specimens as expected for this condition (Fig. 6d–f and Fig. 6a–c, respectively). RhoA (blue) and β-catenin (red) were present, albeit differentially expressed between IgAN and control. While the expression of RhoA (blue) was appreciable in all samples, IgA patients tend to have higher expression, in particular in the glomeruli (most prominent in Fig. 6c versus Fig. 6f). In comparison to healthy biopsies, we observed an increase in β-catenin staining in IgAN glomeruli, most likely in mesangial cells (Fig. 6). Thus, the increase in β-catenin expression might be related to increased Wnt/β-catenin signaling in IgAN glomeruli and this might be related to the deposition of IgA.

## Discussion

In this paper, we present an open-source causal network contextualization tool, CARNIVAL. It seamlessly integrates the information from gene expression data with various types of prior biological knowledge (signaling networks, TF-targets, and pathway-footprints). CARNIVAL merges TF and pathway activities estimated from gene expression data with prior knowledge on the signaling network architecture to identify processes driving changes in gene expression which can be derived from microarray or RNAseq data (see an additional independent study using RNAseq data in Supplementary Text S6). The network inference process is swiftly performed with an ILP formulation of causal reasoning. Importantly, using the variant InvCARNIVAL, the origin of these changes (e.g. perturbation targets) do not need to be known to produce the contextualized networks.

According to our benchmarking study, the introduction of TF weights from DoRothEA improves the performance of StdCARNIVAL (Supplementary Fig. S1). For InvCARNIVAL, a performance was improved with the additional introduction of pathway weights inferred from PROGENy (Supplementary Fig. S2), although the same advantage was not observed in StdCARNIVAL. This demonstrates that the pathway weights only guide the network search if a direction is not provided through a known perturbed target node.

The benchmarking results show that the InvCARNIVAL implementation with TF and pathway weights can obtain perturbation-attributed pathways with a comparable accuracy to StdCARNIVAL (Fig. 3). Therefore, we recommend to include the TF and pathway implementations as the default setting. Comparing to GSEA, perturbation-attributed pathways were more frequently identified with the correct direction. Additionally, a related method, CausalR, did not perform as well as InvCARNIVAL or GSEA in detecting up-regulated pathway sets and was biased towards hub nodes while CARNIVAL was not (Supplementary Fig. S4).

Given that CausalR also captured certain pathways with correct directionality that CARNIVAL missed, results from the two methods could be complementary to each other to ensure the highest coverage of the enriched signaling pathways. It should be noted though that combined results of the two methods at the network level does not increase the number of positive results (see Supplementary Fig. S3). In addition, CARNIVAL offers additional information from its constituting tools (Supplementary Text S4). We therefore propose to also perform functional analyses with DoRothEA and PROGENY along CARNIVAL to get a broader overview for further interpretation at multiple granularities.

In the application study in IgAN, besides standard enrichment analyses, quantitative measures from network topology analyses also pointed to the involvement of multiple signaling pathways in the pathophysiology of the disease including MAPK, neurotrophin, PI3K/Akt, cell cycle, Wnt, and TGF-β pathways. The first two pathways had already been well studied and validated.^26,30^ In our study, we chose the latter two which have less literature support and have inconsistent results to GSEA for experimental validation. Wnt signaling was reported as a dysregulated process in CARNIVAL and is known to be involved in podocyte injury and renal fibrosis.^22^ The IgAN network included representative mediators of the classical Wnt signaling pathway from the messenger WNT7A to the TFs TCF4 and TCF7, although it should be noted that not all of these are linked in the expected ways nor do all members show the expected activity. GSK3B, which is one of the components in the Wnt signaling pathway, also appears among the nodes with top network topology measure, highlighting the importance of this molecule as an important mediator in the signaling regulation of IgAN’s pathophysiology.

TGF-β signaling is a main driver of fibrosis.^21^ CARNIVAL’s IgAN network captured all members of the TGF-β/RhoA pathway as up-regulated and linked through the biologically expected interactions. This includes the TGF-β receptors (TGFBR1 and TGFBR2), the ras homolog gene family member A (RHOA) and the Rho-associated protein kinase 2 (ROCK2). This is consistent with the previously reported upregulation of protein levels of TGF-β receptors and RhoA in IgAN^31,32^ and illustrates how CARNIVAL can identify highly relevant and specific processes and regulators from gene expression data. Our validation experiment shows that both β-catenin and RhoA are more expressed in IgAN compared to healthy controls, consistent with the involvement of these cells in the pathophysiology of the disease. This was not captured via differential expression analysis at the individual gene level and the results were inconclusive via GSEA at the pathway scale (Fig. 6^20^). β-catenin and Wnt signaling are studied as drug targets for different cancers.^33^ They have been proposed as a potential target for chronic kidney.^34^ There are multiple Wnt signaling small-molecule drugs that bind β-catenin.^35^ Such drugs have not been tested in IgAN thus far. Given the pathological importance of the glomerulus in IgAN, the possible differential expression and signaling function of RhoA and Wnt/β-catenin needs to be investigated further. Other dysregulated signaling molecules identified by CARNIVAL, including MAPK14, GSK3B, and CDK5, are also interesting targets for further validation, as their role on the pathophysiology of IgAN is unknown.

Overall, we demonstrate the superior performance of CARNIVAL over existing methods in the benchmarking study and also its applicability to biomedical data in our IgAN case study. Many components in the pipeline are also customizable such as the optimization parameters and the number and selection method of TF inputs. It should be noted though that the benchmarking is performed at the cellular process level due to the limited information on protein activities (see Methods). Moreover, the two-step inference approach also has a few limitations: (1) not all attributed pathways in the KEGG database are represented in MSigDB nor equally relevant, (2) the majority of the KEGG gene sets for canonical pathways do not account for directionality, and (3) gene sets for the same process can be inconsistent in different databases while some are not directly associated with the perturbations. All of these factors could affect the benchmarking results across all methodologies being tested and resulting in lower yield of overall positive results. Further analyses should be performed to determine the generality of our findings.

Although we demonstrated that incorporating prior knowledge into the network inference can lead to a higher accuracy, the drawback is the inherent bias towards known biology. CARNIVAL only uses the known interactions as a scaffold and the contextualization of the network is data driven. Hence, it can still be applied to predict the status of proteins and their connections for specific contexts. Since CARNIVAL cannot propose de novo connections between signaling molecules, it could be combined with pure data-driven network inference approaches such as nested effect models (NEMs)^36^ in the future.

To conclude, we believe that, given the flexibility of the CARNIVAL pipeline, it can be a useful tool to infer context-specific signaling network architectures from gene expression in many studies.

## Methods

### CARNIVAL pipeline

We introduce CARNIVAL, an ILP-based causal network contextualization tool with a high flexibility for data integration. CARNIVAL refines a quantitative objective function for ILP problem by incorporating TF and pathway activities on a continuous scale. In addition, the CARNIVAL framework allows us to contextualize the network with or without known targets of perturbations. The implementation is separated into two pipelines which will be referred henceforth as Standard CARNIVAL “StdCARNIVAL” (with known perturbation targets as an input) and Inverse CARNIVAL “InvCARNIVAL” (without information on targets of perturbation), see Fig. 1.

CARNIVAL is a major improvement and extension of the causal reasoning pipeline by Melas et al. which requires, besides a PKN, discretized differential gene expression, as well as the target(s) of perturbation as inputs. The InvCARNIVAL pipeline overcomes the latter requirement that restricts the original method’s applicability to only well-characterized perturbations. Also, CARNIVAL takes dysregulated TFs derived with DoRothEA^7,11^ which summarizes their activities from the expression of their multiple targets into TF activities with continuous values. This step potentially helps us to reduce the noise from the discretization of individual differential expressed genes and also to reduce the number of measurements inputs which subsequently increase computational efficiency of the pipeline.

### CARNIVAL inputs and data pre-processing

CARNIVAL requires at least two inputs: a PKN consisting of causal protein interactions and a set of measurement inputs derived from gene expression data (either microarray or RNA-seq). The measurement inputs for CARNIVAL are flexible and can be quantitative measures from differentially expressed genes (e.g. log-fold-changes or *t*-values) or predicted TF activities. These measurement inputs are discretized to generate ILP constraints as in the original implementation of Melas et al. The actual continuous input values are used to weight and select causal links in the network. Optionally, predicted pathway activities in a continuous scale between −1 and 1 can also be integrated into CARNIVAL to refine the contextualized network solutions. Targets of perturbation can be provided if known but are not required.

As a PKN, we considered a signed and direct human signaling network retrieved from Omnipath. The network contains 9306 signed and directed edges connecting 3610 nodes pooled and curated from multiple resources including Signor, Reactome, and Wikipathways.^16^ Alternative causal knowledge networks are also compatible with the CARNIVAL pipeline.

For TF activity prediction, we applied DoRothEA version 2^11^ which provides a framework to estimate TF activity from the gene expression of its direct target genes. The provided regulon set was filtered to include only the 289 TF-regulons with at least ten TF-target gene interactions with medium to high confidence (confidence score A, B, and C as defined in^11^). Subsequently, the differential gene expression t-values processed by the *limma* R-package^37^ and the filtered DoRothEA regulon were passed to the viper function in the *VIPER* package^10^ to perform an analytic Rank-based Enrichment Analysis (aREA). The activities of each TF in the form of NES were then derived from the rank of the genes and the top 50 TF scores were used as the input in CARNIVAL by default. Though users can also select the desirable number of included TFs according to the study.

To predict pathway activity, we applied PROGENy, which calculates pathways scores of 14 major signaling pathways based on pathway footprint genes derived from perturbation-based experiments.^7,38^ The 14 PROGENy pathway signatures were obtained from differential expression *t*-values using the *limma* package.^37^ Based on an empirical null distribution generated through 10,000 times gene-wise permutation and the percentile corresponding to the observed value, the significance score (termed “score” henceforth, Eq. 1) was derived:

1 $${\rm{score}}(x) = 2 \cdot ({\rm{percentile}}(x) - 0.5).$$

Regarding known and potential targets of perturbation in the StdCARNIVAL pipeline, users can directly provide the list of targets as being activated, inhibited or unknown (represented by NaN in the R-script). For instance, epidermal growth factor (EGF) is assigned as an “activatory” perturbation target (value of 1) of the experiment with EGF stimulation while phosphoinositide-3-kinase (PI3K) is assigned as an ‘inhibitory’ perturbation target (value of −1) upon the perturbation by its inhibitor such as Wortmannin. The compound target information could be obtained from e.g. the CHEMBL STITCH database via the integrated Omnipath tool.^16^

### CARNIVAL ILP implementation, objective function and parameter settings

We implemented the causal reasoning ILP formulation of Melas et al. (Supplementary Text S1) in R. The objective function is defined by Eq. 2:

2 $${\rm{min}}\left( {\sum \left| \alpha \right| \cdot \left| {x_j - m_j} \right|} \right) + \left( {\sum \beta \cdot (1 - \gamma _i) \cdot x_j^ + + \sum \beta \cdot (1 + \gamma _i) \cdot x_j^ - } \right),$$

where the parameter *α* refers to the mismatch penalty, *β* to the node penalty, and the newly introduced *γ* to the node penalty adjustment (Eq. 2).

In the previous work of Melas et al., the objective function prioritizes the network in which the node activities (*x*_*j*_) explain the corresponding observed discretized measurements (*m*_*j*_) while the overall number of nodes in the network is minimized through the sum of activities $\left( {x_i^ + ,\;x_i^ - } \right)$ for each node *j* in the network. In CARNIVAL, we introduce the effect of inferred TF and pathway activities to adjust this tradeoff and model selection (Eq. 2) where both single samples and differential gene expression can be applied. For TF scores, we applied a TF-specific mismatch penalty *α* corresponding to the NES derived from DoRothEA. The node parameter *β* can then be manually assigned to scale the importance of node penalty relative to TF scores. For pathway scores, a minimal set of representative downstream nodes was chosen for each PROGENy pathway to capture all known signal transduction routes involved while avoiding overlapping information between pathways, and with TF predictions (Supplementary Table S1). The node penalty is sign-adjusted through the *γ* weights which corresponds to PROGENy significance scores (Eq. 1) ranging from −1 to 1. This means that the anticipated direction corresponding to pathway score is penalized less in the expected direction while more in the counterpart.

Regarding parameter settings, we implemented in this study several options to retrieve alternative top scoring solutions through the available CPLEX parameters. The solutions within 0.01% tolerance with regard to the best solution were accepted (mip pool relgap = 0.0001) and the most aggressive search strategy was employed (mip pool intensity = 4). We generated up to 500 solutions (mip limits populate = 500) fulfilling the pre-defined criteria in the solution pool and took the 100 most diverse solutions generated within 1 h for further analysis (mip pool capacity = 100; mip pool replacement = 2; time limit = 3600 s). We applied the default setting for all other CPLEX parameters.

Additional information regarding parameter settings and the ILP problem formulation for InvCARNIVAL can be found in Supplementary Text S2. Summarized results from the study of multiple *α*-to-β ratios to assess parameters’ robustness can be found in Supplementary Text S3.

### Benchmark dataset

The benchmark dataset was taken from the SBVimprover project which contains perturbations on normal human bronchial epithelial cells.^17^ Gene expression of 20 perturbations was measured at 6 h after perturbation (E-MTAB-2091) in a processed form (log2 expression after GC robust multiarray averaging). Probe IDs were mapped to HGNC gene symbols and multiple entries were summarized by the mean value. Batch effects were removed using the combat function of the *sva* R-package.^39^ Differential gene expression compared to vehicle control in the same dataset was then computed with the *limma* R package.^37^

The measurements with 19 phosphoprotein-binding antibodies were mapped to 14 differential protein activities using the curated regulatory sites in the PhosphositePlus knowledgebase.^40^ Given that only a small fraction of the PKN nodes is reported as dysregulated in CARNIVAL, the overlap between dysregulated nodes and measured protein activities was low and not suited for statistical testing.

### Kidney datasets

Microarray data on glomerular gene expression in individual IgAN patients and healthy living donors (HLD) were obtained from five publicly accessible studies,^41–44^ see details in Supplementary Table S2. The effects from covariates embedded in each study and platform represented as batch effects were mitigated using the combat function from the *sva* R-package,^39^ and differential gene expression is determined with the limma R-package.^37^

### CausalR

The CausalR package identifies dysregulated nodes and networks by scanning for nodes with sign-consistent shortest paths to the observations.^13^ With the SCAN (Sequential Causal Analysis of Networks) method, path lengths from one to five edges are scanned and potentially dysregulated nodes are identified which constantly score among the top 150 based on the number of explained observations. Matched observations increase the score ( + 1), mismatched ones decrease it (−1), and unmatched or ambiguously matched nodes are not included in the scoring.

### Gene Set Enrichment Analysis (GSEA)

The *piano* R-package^45^ was applied to run gene set statistics tests using the function *runGSA* with the following methods: *mean, median, sum, maxmean, fisher, stouffer, tailStrength, wilcoxon, page, reporter, and fgsea*. All tests were executed with 10,000 permutations and the *p*-values were adjusted by false discovery rate (FDR).

### Two-step inference approach to KEGG pathways attribution

In our study, we assume that the inferred node activities from CARNIVAL represent upstream signaling and should hence map well with the KEGG pathways attributed to the corresponding perturbation. Hence, a two-step inference approach was developed for validation (Fig. 2). In the first step, we assume that an over-representation of up-regulated CARNIVAL nodes indicates higher activity pathway and, conversely, down-representation represents down-regulation. Up- and down-regulated pathways were predicted with a hypergeometric test from the *Category* package in R on the dysregulated nodes inferred by CARNIVAL. The universe in this regard was set to all nodes present in the PKN derived from Omnipath and the curated KEGG pathway sets were obtained from MSigDB.^46^ A significance test was only performed if at least one set member of the pathway was present in the given CARNIVAL node set.

In the second step, we assumed that the list of KEGG pathways attributed to the perturbation should be up-regulated upon perturbation while others are not. We then check if the pathways identified with CARNIVAL fulfill this assumption (Fig. 2). Specifically, we evaluate if both the up- and down-regulated pathways with their FDR-adjusted *p*-values from the first step were enriched in the attributed pathways. Here, we applied the function *runGSA* with the *stouffer, tailStrength, wilcoxon and reporter* methods in the *piano* R-package.^45^ The mean and standard deviation across the four methods of the resulting *p*-values were reported. A Gene Set Enrichment Analysis (GSEA) was applied to gene expression directly to identify a baseline performance.

### Network topological analyses and text mining

To identify central nodes with regulatory features, we computed quantitative measures from topological analyses of CARNIVAL networks and compare them to the ones derived from randomized networks. These measures were extracted using the functions *degree, betweenness, hub_score and authority_score* in the *igraph* R-package.^47^ The average nodes’ activities over the range of size penalty parameter (β in (0.03; 0.1; 0.3; 0.5; 0.8)) in the CARNIVAL networks were applied as weights for the calculation of these topological analyses measures. The R-package *BiRewire*^48^ was applied to generate 100 randomized networks with preserved distributions of node degrees as well as interaction signs. Student’s *t* test was subsequently used to determine significant differences between the distributions of network topology measures from CARNIVAL networks versus the ones from randomized networks. To assess the “novelty” of genes identified in this analysis in the context of IgA nephropathy we queried PubMed Central using the approved gene symbol and approved gene name from the HUGO gene nomenclature committee database (genenames.org), together with the following keywords: “IgA nephropathy”, “IgAN”, “IgA glomerulonephritis” and “Berger’s disease”. All search terms were enclosed in quotations to ensure that terms were searched in full and retrieved abstracts were examined to exclude erroneous matches.

### Statistical analyses and data representation

Student’s *t* tests were performed to identify statistical differences between two groups using the function *t.test* in the *stats* R-package. Unpaired two-sided *t*-tests with non-equal variance was applied by default unless specified. Pearson and Spearman correlation measures were calculated using the function *cor* in the *stats* R-package. Jaccard similarity index is defined as the ratio between the size of the intersection and the size of the union of two sets. Boxplots represent the interquartile range (IQR) with center lines represent median of minus log10 *p*-values. Outliers are defined by the data points below the first quartile minus 1.5*IQR and above the third quartile plus 1.5*IQR. CARNIVAL networks were exported to Cytoscape to generate figures for publication.^49^ No Bayesian analysis nor hierarchical and complex designs was performed in this study.

### Reporting summary

Further information on experimental design is available in the Nature Research Reporting Summary linked to this paper.

## Supplementary information

Supplementary materials

Nature Report: Reporting summary

## Data Availability

The benchmark dataset was taken from Poussin et al.^17^ which is available on ArrayExpress with accession code “E-MTAB-2091”. The data sources of the kidney dataset for IgAN patients and healthy samples were derived from several studies.^41–44^ These datasets are publicly available on Gene Expression Omnibus (GEO) repository with the accession codes “GSE37460”, “GSE50469”, “GSE93789”, “GSE30122” and “GSE32591” as summarized in Supplementary Table S2.

## Code availability

CARNIVAL is publicly available as an R-package on GitHub through the following URL: https://github.com/saezlab/CARNIVAL.
