# Supplementary material for: From expression footprints to causal pathways: contextualizing large signaling networks with CARNIVAL
Source: NPJ Syst Biol Appl. 2019 Nov 11;5:40. doi: 10.1038/s41540-019-0118-z (PMC6848167; doi:10.1038/s41540-019-0118-z)
Supplement: Supplementary file 1 — Supplementary materials [file 41540_2019_118_MOESM1_ESM.pdf]

Supplementary Information:

From expression footprints to causal pathways: contextualizing large signaling networks with CARNIVAL

Anika Liu[1,2,*], Panuwat Trairatphisan[1,*], Enio Gjerga[1,2,*], Athanasios Didangelos[3], Jonathan Barratt[3], Julio Saez-Rodriguez[1,2,#]

[1] Heidelberg University, Faculty of Medicine, Institute of Computational Biomedicine, 69120 Heidelberg, Germany
[2] RWTH Aachen University, Faculty of Medicine, Joint Research Centre for Computational Biomedicine (JRC-COMBINE), 52074, Aachen, Germany
[3] Department of Infection, Immunity and Inflammation, University of Leicester, Leicester, UK

* co-first authors
[#] Corresponding author: julio.saez@bioquant.uni-heidelberg.de

Supplementary Figures:

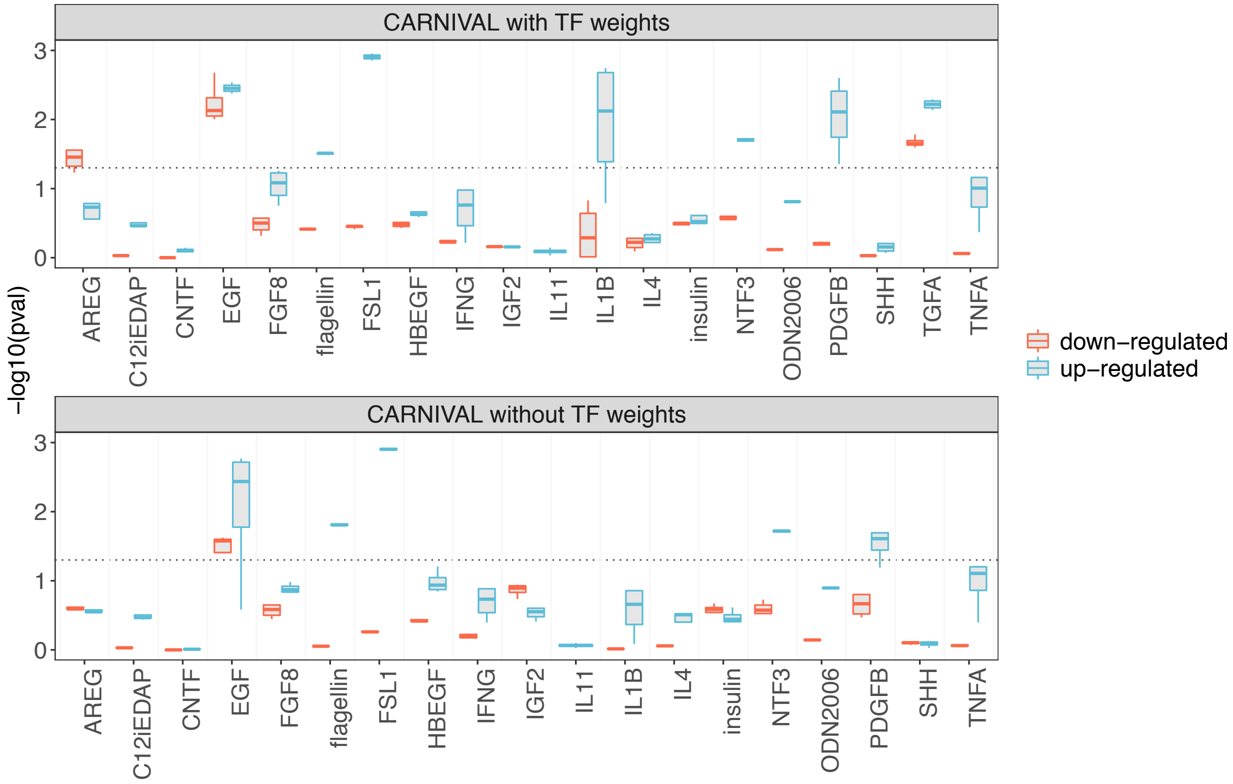

Figure S1. Results from with and without the integration of TF weights in standard CARNIVAL. Results are shown for beta = 1.

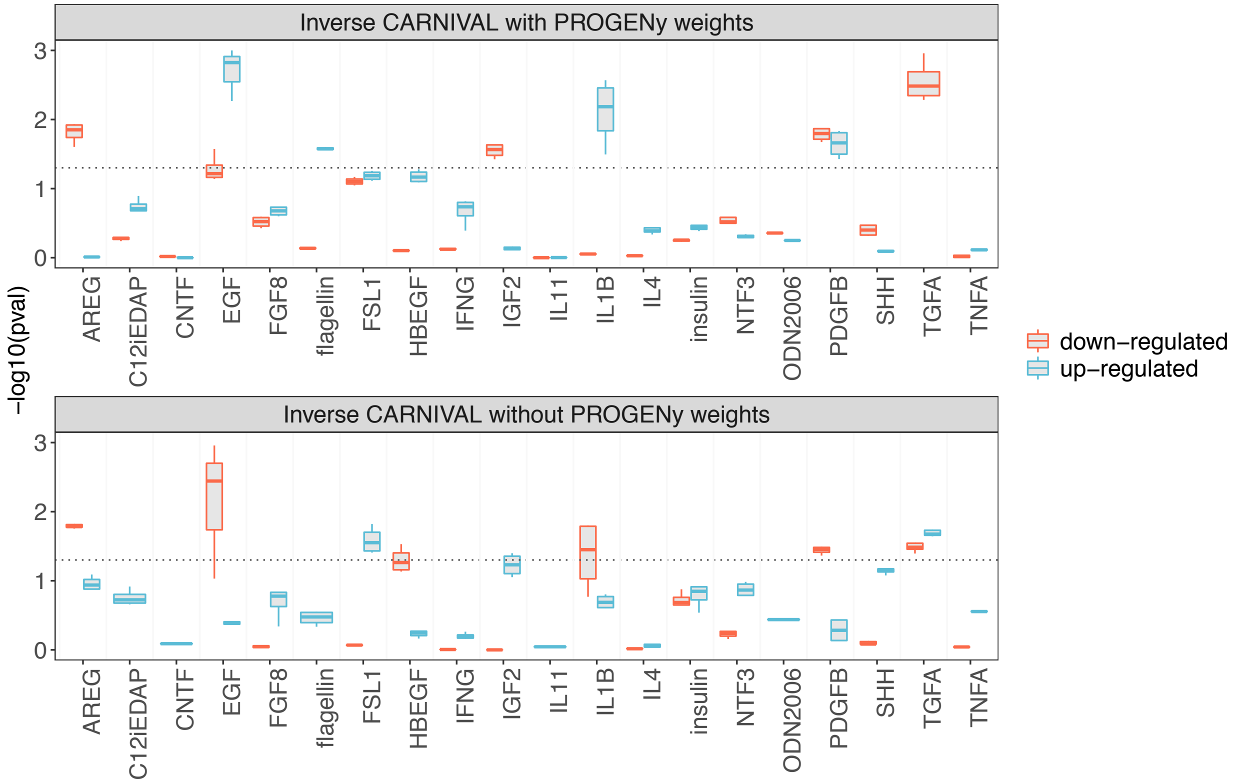

Figure S2. Results from the integration of pathway weights in inverse CARNIVAL. Results are shown for beta = 0.5.

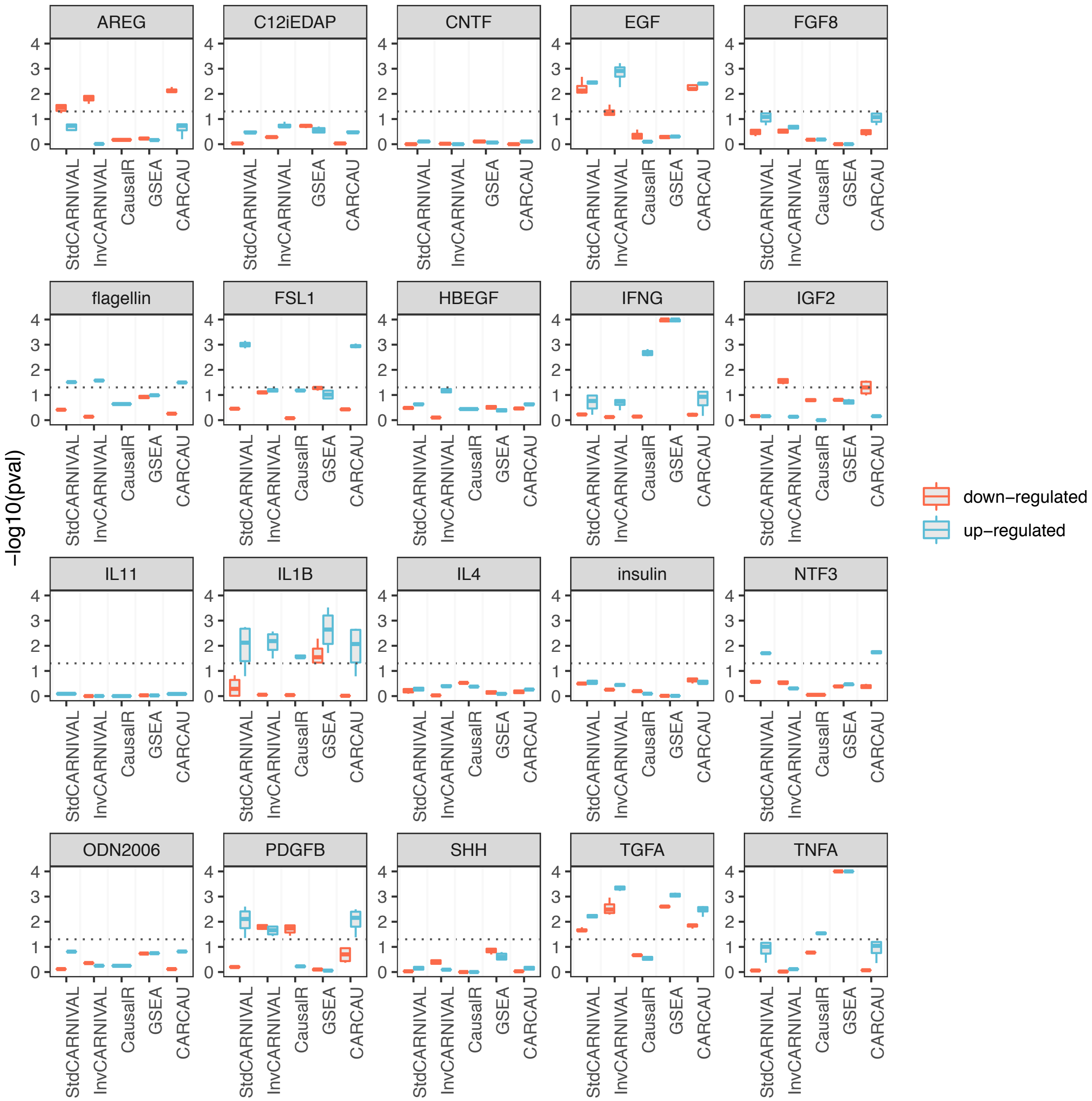

Figure S3. Comparison of the enrichment results of the perturbation-attributed pathway set in dysregulated pathways inferred with different tools. An enrichment of the perturbation-attributed pathway set among the significant pathways was determined. For GSEA, dysregulated pathways were inferred with the *piano* R-package to determine whether a certain pathway is activated (up-regulated) or inhibited (down-regulated). For CausalR and CARNIVAL, an over-representation analysis of up- or down-regulated solution nodes in the KEGG gene sets was used as a proxy instead. The combined results from StdCARNIVAL and CausalR are labeled as “CARCAU”. The significance level of 0.05 is indicated by the dotted lines.

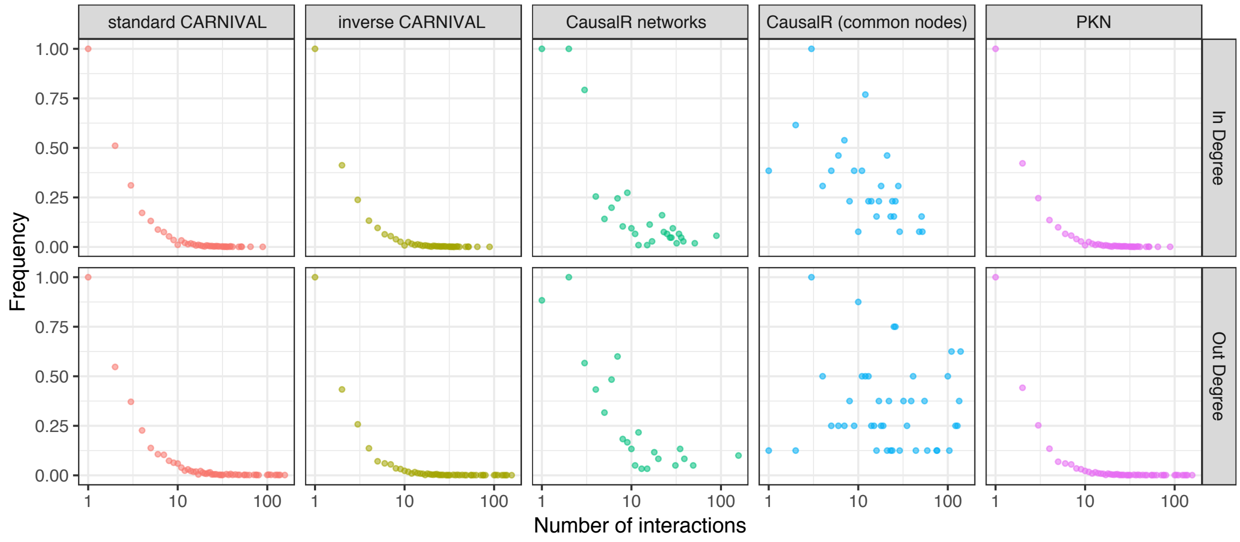

Figure S4. Comparison of node connectivity distributions for networks from CARNIVAL and CausalR comparing to the prior knowledge network (PKN).

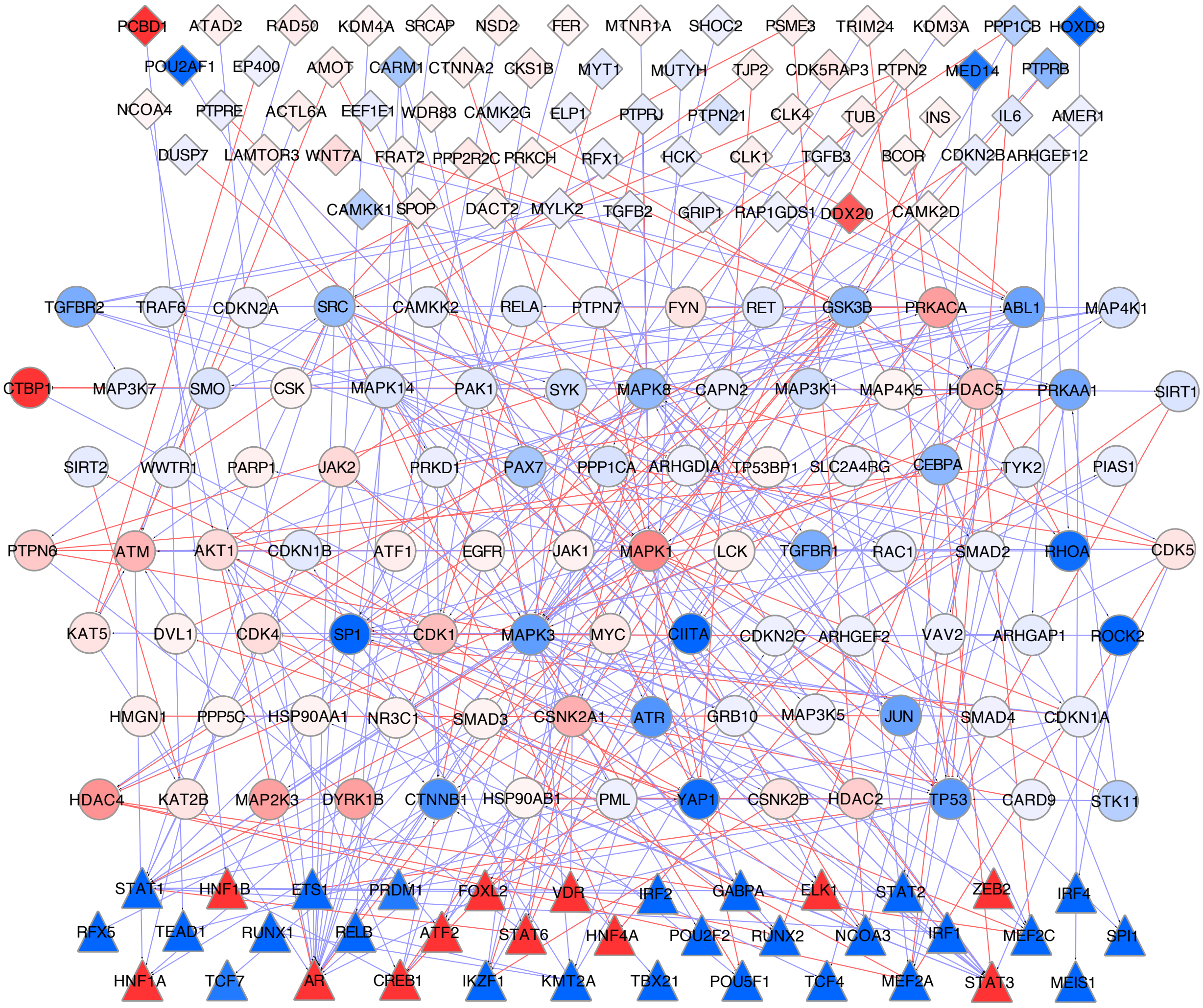

Figure S5. Combined IgAN-contextualized networks from CARNIVAL for all node penalties β in (0.03; 0.1; 0.3; 0.5; 0.8). Up-regulated nodes and activatory reactions are indicated in blue while down-regulated nodes and inhibitory edges are colored in red. Triangles correspond to transcription factors, diamonds represent input nodes and circles correspond to purely inferred nodes. The intensity of color refers to the average node activities from all node penalties.

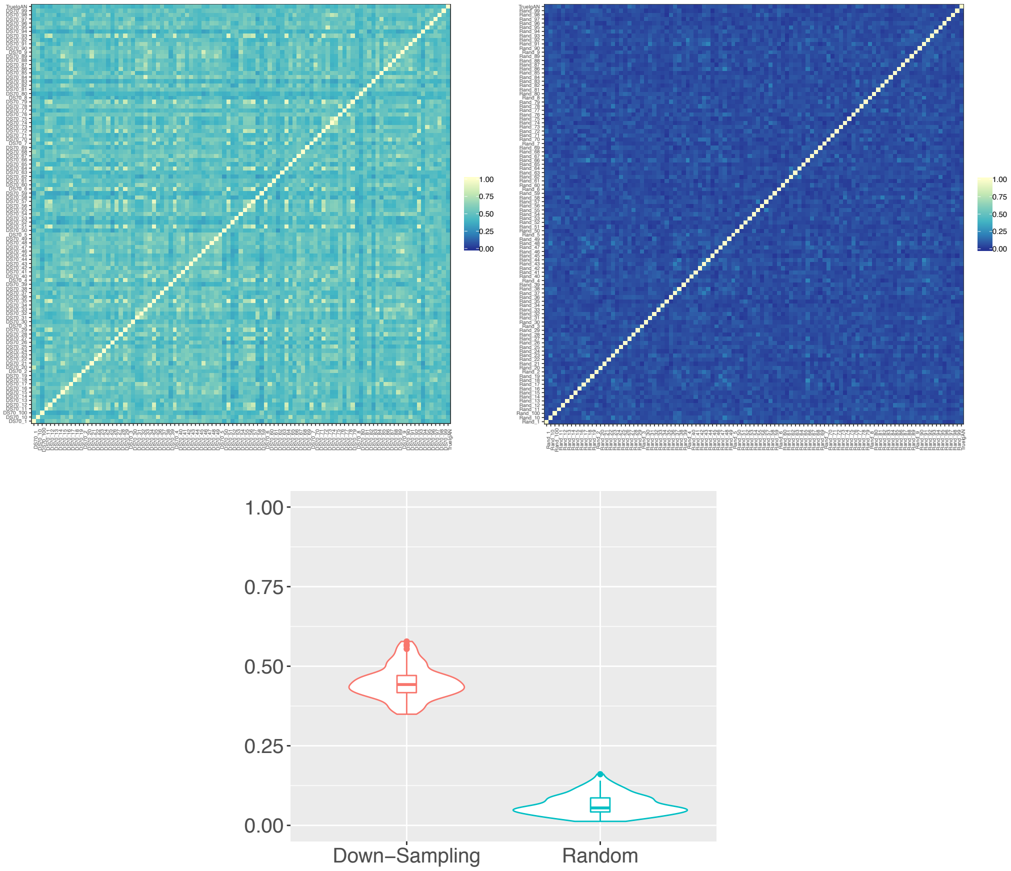

Figure S6. Jaccard similarity measures of 100 networks generated by 70% down-sampling (top-left) and by re-shuffling of transcription factor (TF) inputs' labels (top-right) compared to the true IgAN network from actual data. The mean and standard deviation of the Jaccard similarity measure of the 70% down-sampling networks is 0.448 +/- 0.051 (mean +/- S.D.) while they are 0.065 +/- 0.032 (mean +/- S.D.) from TF-labels reshuffling (bottom). The p-value from one-sided Komogorov-Smirnov test between the two sets of Jaccard similarity distribution is 3.72e-44.

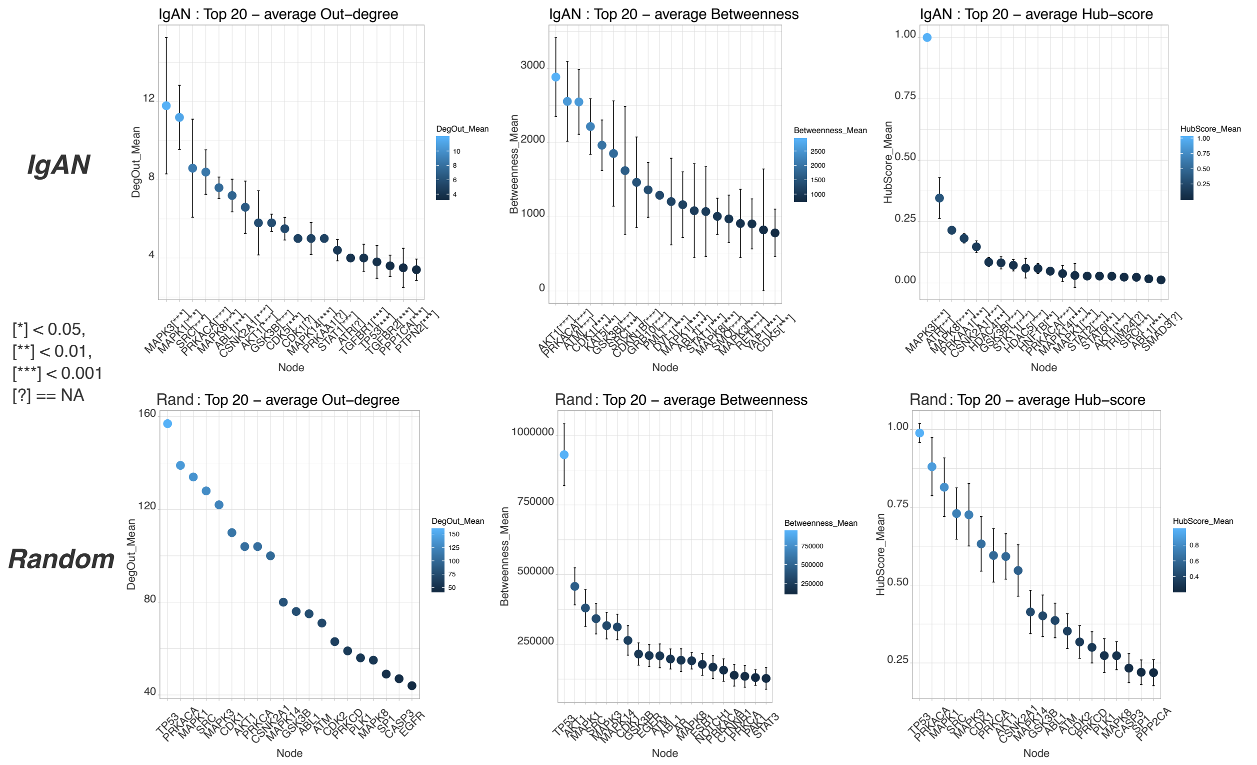

Figure S7. Network topology measures from IgAN-contextualized networks from CARNIVAL at five different size penalty (beta=0.03,0.1,0.3,0.5,0.8) and from 100 randomized networks with degree and sign preserved generated from the prior knowledge network. Dots and error bars represent the mean and standard deviation for each node in each category. Significant differences from t-test are highlighted with asterisks (*) after the protein names in the IgAN set. Question marks (?) were annotated when a significant test could not performed e.g. due to limited sample sizes.

Supplementary Tables:

Table S1. Representative nodes of PROGENy pathway in CARNIVAL. A minimal number of representative nodes were chosen in a way that covers all associated signalling routes described in KEGG while avoiding overlaps between pathways and with DoRothEA TFs if possible.

Pathway	Representative nodes
EGFR	EGFR, ERBB2
Hypoxia	HIF1A
JAK/STAT	JAK1, JAK2, JAK3
MAPK	BRAF, ARAF, RAF1
NFkB	NFKB1
PI3K	PIK3CA, PIK3CB, PIK3CD, PIK3CG
TGFb	TGFBR1, TGFBR2, BMPR1A, BMPR1B, BMPR2
TNFa	TNFRSF1A, TNFRSF1B
Trail	CASP8, CASP10
VEGF	FLT1, FLT3, KDR, PDGFRA, PDGFRB
p53	TP53
Androgen	AR
Estrogen	ESR1, ESR2
WNT	DVL1

Table S2. Glomerular gene expression datasets. Microarray gene expression data for IgAN patients and healthy living donors (HLD) were accessed from the GEO database[1]. The accession number is shown together with the number of samples in each class (IgAN, HLD).

Study	GEO accession	Platform	HLD	IgAN
Berthier et al. (2012)[2]	GSE37460	GPL96 & GPL570	27	27
Hodgin et al. (2014)[3]	GSE50469	GPL96 & GPL570	0	22
Liu et al. (2017)[4]	GSE93789	GPL570	22	20

Woroniecka et al. (2011)[5]	GSE30122	GPL571	6	0
Berthier et al. (2012)[2]	GSE32591	GPL96	4	0

Table S3. Top 20 nodes and edges with highest average network topology measures from the IgAN networks with different node penalty parameters (β in (0.03; 0.1; 0.3; 0.5; 0.8), see Methods).

Top 20 In-degree						
Protein	In-degree	Out-degree	All-degree	Betweenness	Hub-score	Authority-score
AR	**8.2**	0	8.2	0	0.0000	0.0095
TP53	**7.4**	3.8	11.2	609.2	0.0024	1.0000
STAT3	**7.2**	0.4	7.6	135.3	0.0000	0.0302
MAPK1	**6.4**	11.2	17.6	1164.2	0.0308	0.0002
STAT1	**6.4**	4.4	10.8	1071.7	0.0001	0.1163
MAPK8	**5.6**	7.6	13.2	1007.1	0.2150	0.0000
ATM	**5.2**	3	8.2	2550	0.0027	0.0002
GSK3B	**5.2**	5.8	11	1854.6	0.0821	0.0142
CDKN1A	**5**	1	6	170.125	0.0000	0.0056
CREB1	**5**	0.2	5.2	22	0.0000	0.2396
MEF2A	**5**	0	5	0	0.0000	0.0453
CDK1	**4.8**	5	9.8	2218.3	0.0016	0.0040
MEF2C	**4.8**	0	4.8	0	0.0000	0.0492
SP1	**4.8**	1.8	6.6	76.4	0.0001	0.9537
ABL1	**4.6**	7.2	11.8	1082.8	0.0170	0.0060
IKZF1	**4.6**	0	4.6	0	0.0000	0.0522
SRC	**4.4**	8.6	13	1623.6	0.0234	0.0020
STAT6	**4.2**	1	5.2	137.6	0.0282	0.0589
YAP1	**4.2**	2.6	6.8	824.1	0.0023	0.0137
CTNNB1	**3.8**	2	5.8	86.2	0.0000	0.0083
Top 20 Out-degree						
Protein	In-degree	Out-degree	All-degree	Betweenness	Hub-score	Authority-score
MAPK3	3	**11.8**	14.8	910.5	1.0000	0.0000
MAPK1	6.4	**11.2**	17.6	1164.2	0.0308	0.0002
SRC	4.4	**8.6**	13	1623.6	0.0234	0.0020
PRKACA	2	**8.4**	10.4	2557.4	0.0483	0.0079
MAPK8	5.6	**7.6**	13.2	1007.1	0.2150	0.0000
ABL1	4.6	**7.2**	11.8	1082.8	0.0170	0.0060
CSNK2A1	1	**6.6**	7.6	195.4	0.1474	0.0124
AKT1	3.4	**5.8**	9.2	2885.6	0.0277	0.0057
GSK3B	5.2	**5.8**	11	1854.6	0.0821	0.0142
CDK5	1	**5.5**	6.5	783.5	0.0014	0.0000

CDK1	4.8	**5**	9.8	2218.3	0.0016	0.0040
MAPK14	1	**5**	6	263.125	0.0382	0.0000
PRKAA1	2.4	**5**	7.4	114.4	0.1819	0.0160
STAT1	6.4	**4.4**	10.8	1071.7	0.0001	0.1163
ATR	2.8	**4**	6.8	324	0.3459	0.0059
TGFBR1	1.6	**4**	5.6	68.8	0.0018	0.0000
TP53	7.4	**3.8**	11.2	609.2	0.0024	1.0000
TGFBR2	1.6	**3.6**	5.2	337	0.0000	0.0068
PPP1CA	1.25	**3.5**	4.75	631.25	0.0021	0.0002
PTPN2	0	**3.4**	3.4	0	0.0037	0.0000
Top 20 All-degree						
Protein	In-degree	Out-degree	All-degree	Betweenness	Hub-score	Authority-score
MAPK1	6.4	11.2	**17.6**	1164.2	0.0308	0.0002
MAPK3	3	11.8	**14.8**	910.5	1.0000	0.0000
MAPK8	5.6	7.6	**13.2**	1007.1	0.2150	0.0000
SRC	4.4	8.6	**13**	1623.6	0.0234	0.0020
ABL1	4.6	7.2	**11.8**	1082.8	0.0170	0.0060
TP53	7.4	3.8	**11.2**	609.2	0.0024	1.0000
GSK3B	5.2	5.8	**11**	1854.6	0.0821	0.0142
STAT1	6.4	4.4	**10.8**	1071.7	0.0001	0.1163
PRKACA	2	8.4	**10.4**	2557.4	0.0483	0.0079
CDK1	4.8	5	**9.8**	2218.3	0.0016	0.0040
AKT1	3.4	5.8	**9.2**	2885.6	0.0277	0.0057
AR	8.2	0	**8.2**	0	0.0000	0.0095
ATM	5.2	3	**8.2**	2550	0.0027	0.0002
CSNK2A1	1	6.6	**7.6**	195.4	0.1474	0.0124
STAT3	7.2	0.4	**7.6**	135.3	0.0000	0.0302
PRKAA1	2.4	5	**7.4**	114.4	0.1819	0.0160
ATR	2.8	4	**6.8**	324	0.3459	0.0059
YAP1	4.2	2.6	**6.8**	824.1	0.0023	0.0137
HDAC5	3.6	3	**6.6**	36.6	0.0604	0.0379
SP1	4.8	1.8	**6.6**	76.4	0.0001	0.9537
Top 20 Betweenness						
Protein	In-degree	Out-degree	All-degree	Betweenness	Hub-score	Authority-score
AKT1	3.4	5.8	9.2	**2885.6**	0.0277	0.0057
PRKACA	2	8.4	10.4	**2557.4**	0.0483	0.0079
ATM	5.2	3	8.2	**2550**	0.0027	0.0002
CDK1	4.8	5	9.8	**2218.3**	0.0016	0.0040
KAT5	1.4	2	3.4	**1966.5**	0.0003	0.0001
GSK3B	5.2	5.8	11	**1854.6**	0.0821	0.0142
SRC	4.4	8.6	13	**1623.6**	0.0234	0.0020
CDKN1B	2	1	3	**1466**	0.0001	0.0000
GRB10	2	1	3	**1364**	0.0000	0.0159

PML	2	1	3	**1291.25**	0.0001	0.0252
DVL1	1	1	2	**1206.166667**	0.0000	0.0000
MAPK1	6.4	11.2	17.6	**1164.2**	0.0308	0.0002
ABL1	4.6	7.2	11.8	**1082.8**	0.0170	0.0060
STAT1	6.4	4.4	10.8	**1071.7**	0.0001	0.1163
MAPK8	5.6	7.6	13.2	**1007.1**	0.2150	0.0000
SMO	1	1	2	**972.625**	0.0001	0.0054
MAPK3	3	11.8	14.8	**910.5**	1.0000	0.0000
RET	1	2	3	**905.125**	0.0000	0.0025
YAP1	4.2	2.6	6.8	**824.1**	0.0023	0.0137
CDK5	1	5.5	6.5	**783.5**	0.0014	0.0000
Top 20 Hub-score						
Protein	In-degree	Out-degree	All-degree	Betweenness	Hub-score	Authority-score
MAPK3	3	11.8	14.8	910.5	**1.0000**	0.0000
ATR	2.8	4	6.8	324	**0.3459**	0.0059
MAPK8	5.6	7.6	13.2	1007.1	**0.2150**	0.0000
PRKAA1	2.4	5	7.4	114.4	**0.1819**	0.0160
CSNK2A1	1	6.6	7.6	195.4	**0.1474**	0.0124
HDAC4	2.8	3	5.8	57.7	**0.0851**	0.0497
GSK3B	5.2	5.8	11	1854.6	**0.0821**	0.0142
STK11	1	3	4	417.4	**0.0722**	0.0067
HDAC5	3.6	3	6.6	36.6	**0.0604**	0.0379
HNF1B	1	1.8	2.8	73.2	**0.0588**	0.0000
PRKACA	2	8.4	10.4	2557.4	**0.0483**	0.0079
MAPK14	1	5	6	263.125	**0.0382**	0.0000
MAPK1	6.4	11.2	17.6	1164.2	**0.0308**	0.0002
STAT2	1.4	1	2.4	71.6	**0.0282**	0.0001
STAT6	4.2	1	5.2	137.6	**0.0282**	0.0589
AKT1	3.4	5.8	9.2	2885.6	**0.0277**	0.0057
TRIM24	0	2	2	0	**0.0239**	0.0000
SRC	4.4	8.6	13	1623.6	**0.0234**	0.0020
ABL1	4.6	7.2	11.8	1082.8	**0.0170**	0.0060
SMAD3	1	3	4	46	**0.0126**	0.0000
Top 20 Authority-score						
Protein	In-degree	Out-degree	All-degree	Betweenness	Hub-score	Authority-score
TP53	7.4	3.8	11.2	609.2	0.0024	**1.0000**
SP1	4.8	1.8	6.6	76.4	0.0001	**0.9537**
RUNX2	3.4	0	3.4	0	0.0000	**0.7359**
GABPA	1.2	0	1.2	0	0.0000	**0.6918**
ETS1	1.2	0	1.2	0	0.0000	**0.6910**
JUN	3.8	2.2	6	111.1	0.0000	**0.4111**
CREB1	5	0.2	5.2	22	0.0000	**0.2396**
KMT2A	2	3	5	380.2	0.0001	**0.1566**

PTPN6	1.4	2.2	3.6	175.8	0.0030	**0.1449**
CTBP1	2	1	3	69.4	0.0000	**0.1371**
JAK2	1	2	3	23.2	0.0039	**0.1284**
STAT1	6.4	4.4	10.8	1071.7	0.0001	**0.1163**
HNF4A	2	0	2	0	0.0000	**0.0865**
VDR	2.2	0	2.2	0	0.0000	**0.0689**
STAT6	4.2	1	5.2	137.6	0.0282	**0.0589**
NR3C1	2	1	3	18	0.0000	**0.0534**
IKZF1	4.6	0	4.6	0	0.0000	**0.0522**
HDAC4	2.8	3	5.8	57.7	0.0851	**0.0497**
MEF2C	4.8	0	4.8	0	0.0000	**0.0492**
MEF2A	5	0	5	0	0.0000	**0.0453**

Table S4. Results from literature search based on the list of up- and down-regulated nodes identified by CARNIVAL. 'Hits' refers to the number of these nodes being present together with the IgAN term. The suffixes '_up' and '_dn' refers to the activity of the nodes in CARNIVAL networks. To account for consistencies, only the nodes which are present in at least 4 out of 5 size penalty parameters (beta) being tested are included. No conflicting CARNIVAL node's activity was observed.

Hits	CARNIVAL nodes
8	JUN_up
7	MAPK1_dn, MAPK3_up, SYK_up
6	STAT3_dn
4	CDK1_dn, MAPK8_up
3	AKT1_dn, CTNNB1_up, MYC_dn
2	CARD9_up, GABPA_up, JAK2_dn, RHOA_up, SP1_up, SRC_up, STAT1_up, STAT6_dn, VDR_dn, ZEB2_dn
1	ATF1_dn, CDKN1A_up, CDKN1B_up, HDAC2_dn, HDAC4_dn, KMT2A_up, PRKACA_dn, TBX21_up, TP53_up, TRAF6_up
0	ABL1_up, ACTL6A_dn, AR_dn, ARHGAP1_up, ARHGEF12_up, ATAD2_dn, ATF2_dn, ATM_dn, ATR_up, CAMKK1_up, CARM1_up, CDK4_dn, CDK5_dn, CDK5RAP3_dn, CDKN2B_up, CEBPA_up, CIITA_up, CKS1B_dn, CLK1_dn, CREB1_dn, CSNK2A1_dn, CSNK2B_dn, CTBP1_dn, DDX20_dn, DUSP7_up,

	DYRK1B_dn, EEF1E1_up, ELK1_dn, ETS1_up, FOXL2_dn, FYN_dn, GSK3B_up, HCK_up, HDAC5_dn, HNF1A_dn, HNF1B_dn, HNF4A_dn, HOXD9_up, IKZF1_up, IRF1_up, IRF2_up, IRF4_up, KAT2B_dn, KAT5_dn, LAMTOR3_dn, MAP2K3_dn, MAP3K1_up, MAP3K7_up, MAP4K1_up, MAPK14_up, MED14_up, MEF2A_up, MEF2C_up, MEIS1_up, MTNR1A_dn, MUTYH_up, MYT1_up, NCOA3_up, PAX7_up, PCBD1_dn, POU2AF1_up, POU2F2_up, POU5F1_up, PPP1CA_up, PPP1CB_up, PPP2R2C_dn, PRDM1_up, PRKAA1_up, PTPN21_up, PTPN6_dn, PTPRB_up, PTPRJ_up, RAD50_dn, RELB_up, RET_up, RFX1_up, RFX5_up, ROCK2_up, RUNX1_up, RUNX2_up, SIRT2_up, SMO_up, SPI1_up, STAT2_up, STK11_up, TCF4_up, TCF7_up, TEAD1_up, TGFBR1_up, TGFBR2_up, WNT7A_dn, YAP1_up

Table S5. Comparison of p-values from the two-step inference analysis of CARNIVAL and GSEA results. The p-values of CARNIVAL results are the average values from the CARNIVAL results with different node penalty parameters (β in (0.03; 0.1; 0.3; 0.5; 0.8), see Methods).

Pathway	CARNIVAL		GSEA	
	Up	Down	Up	Down
ADHERENS_JUNCTION	3.97E-07	0.005333067	3.32E-07	0.000477927
TGF_BETA_SIGNALING_PATHWAY	0.001361965	0.417998022	7.55E-05	0.003303530
TIGHT_JUNCTION	0.230886127	0.001114261	0.221616881	8.40E-05
FOCAL_ADHESION	0.000289652	0.287633245	0.000211657	0.226049943
NEUROTROPHIN_SIGNALING_PATHWAY	0.0004987	0.508766561	0.000426883	0.347757834
MAPK_SIGNALING_PATHWAY	0.000571613	0.05861374	0.000426327	0.034538337
WNT_SIGNALING_PATHWAY	0.001277748	0.020018325	0.001512523	0.002791867
VASCULAR_SMOOTH_MUSCLE_CONTRACTION	0.007026133	0.222632998	0.001953213	0.112084215
ERBB_SIGNALING_PATHWAY	0.002337064	0.08835585	0.001873277	0.044712518
CELL_CYCLE	0.027777764	0.066435369	0.00204617	0.035192772

Supplementary Texts:

Text S1. ILP implementation.

We re-implemented the integer linear programming (ILP) formulation of causal reasoning problem presented in Melas et al.[6] in the R programming language. A summarized description of the ILP formation is shown as follows:

A signaling network G is an interaction graph defined by a set of signed and directed reactions $i = 1,2,\ldots,n_r$, and a set of nodes $j = 1,2,\ldots,n_s$. Thereby, each reaction i is characterized by an ordered pair consisting of source species S_i and target species T_i with $S_i, T_i \in \{1,2,\ldots,n_s\}$. The reaction sign is denoted by $\sigma_i \in \{-1,1\}$ and distinguishes between activation ($\sigma_i = 1$) and inhibition ($\sigma_i = -1$). The ILP variable definitions are summarized in Table 1 and provide a key to the used notation.

Table 1. The list of ILP variables and their descriptions

Variable	Description
$u_i^+ \in \{0,1\}$	Potential of reaction i to activate its target node
$u_i^- \in \{0,1\}$	Potential of reaction i to inhibit its target node
$\sigma_i \in \{-1,1\}$	Sign of reaction i
$x_j^+ \in \{0,1\}$	Potential of node j to be activated
$x_j^- \in \{0,1\}$	Potential of node j to be inhibited
$x_j \in \{-1,0,1\}$	Predicted activation/inhibition state of species j
$m_j \in \{-1,0,1\}$	Activation/inhibition state of measured species j
$I_j \in \{-1,0,1,NaN\}$	Activation/inhibition state of perturbed species j; NaN represents the unknown state
$B_j \in \{-1,0,1\}$	Auxiliary variable to determine the state perturbed species j once the input value $I_j = NaN$
$d_j \in [0, M]$	Auxiliary distance variables assigned to each node j where $M$is a sufficiently large number (default: $M = 100$)
$A_j \in [0,2]$	Auxiliary variable representing the absolute difference between the inferred and measured species j.

The variables in Table 1 are determined during the linear programming optimisation according to the following set of constraints of causal reasoning principle. The activation state of a reaction i is defined by the activity of its source node X_{Si} and the reaction sign σ_i. The reaction has a potential to activate its target node ($u_i^+ = 1$), if and only if $\sigma_i \cdot x_{Si} = 1$. This occurs in two cases: either the source node is activated ($x_j = S_i = 1$) and has an activating effect on its target node ($\sigma_i = 1$); or the source node is inhibited ($x_j = S_i = -1$) and has an inhibiting effect ($\sigma_i = -1$). Vice versa, a reaction has the potential to downregulate its target node ($u_i^- = 1$), if and only if $\sigma_i \cdot x_{Si} = -1$.

$$u_i^+ \geq \sigma_i x_j \text{ where } i \in \{1,2,\ldots,n_r\}\,;\, j \in \{1,2,\ldots,n_s\} \quad \ldots c_1$$
$$u_i^- \geq -\sigma_i x_j \text{ where } i \in \{1,2,\ldots,n_r\}\,;\, j \in \{1,2,\ldots,n_s\} \quad \ldots c_2$$
$$u_i^+ \leq 1 - u_i^- \text{ where } i \in \{1,2,\ldots,n_r\} \quad \ldots c_3$$
$$u_i^+ \leq \sigma_i x_j + u_i^- \text{ where } i \in \{1,2,\ldots,n_r\}\,;\, j \in \{1,2,\ldots,n_s\} \quad \ldots c_4$$
$$u_i^- \leq -\sigma_i x_j + u_i^+ \text{ where } i \in \{1,2,\ldots,n_r\}\,;\, j \in \{1,2,\ldots,n_s\} \quad \ldots c_5$$

For the definition of the activation state of a node x_j, two cases can be distinguished: For the non-input nodes, the activity of these nodes is defined by the potentials of incoming reactions. If and only if at least one incoming reaction has the potential to activate ($u_i^+ : T_{i=j} = 1$), the node can have the potential to be activated ($x_j^+ = 0 \vee 1$). Vice versa, a node can only have the potential to be down-regulated ($x_j^- = 0 \vee 1$) if at least one incoming reaction has the potential to inhibit ($u_i^- : T_{i=j} = 1$). A node is then up-regulated ($x_j = 1$), if there is exclusively a potential to be up-regulated ($x_j^+ = 1$), and is down-regulated ($x_j = -1$) if there is exclusively a potential to be down-regulated ($x_j^- = 1$). If none or both of the potentials exist, the node will remain neutral ($x_j = 0$).

$$x_j^+ \leq \sum_{i:T_i=j} u_i^+ \text{ where } i \in \{1,2,\ldots,n_r\}\,;\; j \in \{1,2,\ldots,n_s\} \quad \ldots c_6$$
$$x_j^- \leq \sum_{i:T_i=j} u_i^- \text{ where } i \in \{1,2,\ldots,n_r\}\,;\; j \in \{1,2,\ldots,n_s\} \quad \ldots c_7$$

For perturbed/input nodes, the activation state can be user-defined ($x_j = I_j$). In the case where the perturbed node is known but the activation/inhibition state remains unknown, the notation NaN can be assigned. The state of x_j is equal to B_j in this case and will then take any of the three states $x_j \in \{-1,0,1\}$. All remaining input nodes which were not defined for its activation state in the input list will always take the value 0 and will not influence the system.

$$x_j = x_j^+ - x_j^- + B_j \text{ where } j \in \{1,2,\ldots,n_s\} \quad \ldots c_8\,[1]$$
$$x_j = I_j \text{ where } j \in \mathrm{I}\,;\; I_j \in \{-1,0,1\} \quad \ldots c_8\,[2]$$
$$x_j = B_j \text{ where } j \in \mathrm{S} - \mathrm{T} \quad \ldots c_8\,[3]$$
$$B_j = 0 \text{ where } j \in (\mathrm{S} \cup \mathrm{T}) - I \quad \ldots c_8\,[4]$$

In addition, feedback loops are removed given that effects mediated through those are highly dynamic and hardly interpretable from a static snapshot as in an interaction network. For instance, positive feedback loops can lead to internal signals independent to external perturbations. These were constrained through a distance variable d_j. Thereby, the distance of all nodes connected to a perturbation node is set to a value larger than zero, while all others are defined to be zero. As a consequence, only nodes connected to a perturbation can be deregulated. The distance increases

from source node to target node if the interaction is active, i.e. $u_i^+ = 1 \vee u_i^- = 1$, and is not allowed to pass the distance threshold M, which is considerably larger than expected path lengths.

$$x_i^+ \le d_j \quad \ldots c_l\ [1]$$
$$x_i^- \le d_j \quad \ldots c_l\ [2]$$
$$d_{T_i} \ge d_{S_i} + 1 - M + u_i^+ M \quad \ldots c_l\ [3]$$
$$d_{T_j} \ge d_{S_i} + 1 - M + u_i^- M \quad \ldots c_l\ [4]$$
$$d_j \le M \quad \ldots c_l\ [5]$$

Text S2. Additional parameter settings and the inverse CARNIVAL pipeline.

It should be noted that, while the objective function is able to rank network solutions according to the pre-defined criteria as described in the manuscript, this does not imply that the best-scoring network solutions from each parameter setting need to be similar. We therefore also explored the results generated from multiple α-to-β ratios. While larger node penalties frequently did not produce solutions, a node penalty between 0.03 and 1.5 resulted in similar results and similar performance for standard CARNIVAL. Given that inverse CARNIVAL was found to be more sensitive towards changes in node penalty, a value between 0.03 and 0.5 is recommended for inverse CARNIVAL. Summarised results from the study of multiple α-to-β ratios can be found in Supplementary Text S3.

The inverse CARNIVAL pipeline allows network inference without protein target information. While the ILP formulation itself requires known target proteins, a list of all potential perturbed nodes can be directly generated from the prior knowledge network, and can be used to capture all potential paths. In this study, we generated this list from all nodes with only outgoing and no incoming edges.

In the original ILP formulation, input nodes are not subjected to the node penalty and were already pre-defined with regard to their activity state. In inverse CARNIVAL, even if the activity state of the inputs are unknown, an input node penalty is still desired to limit the number of input nodes in the solution network. This can be achieved by adding an extra perturbation node to the prior knowledge network which has both outgoing activating and inhibiting edges to all potential input nodes. This perturbation node then serves as an unpenalized target of perturbation and can be linked to all potential input nodes without restricting their activity state. These potential input nodes are then penalized as normal additional nodes if deregulated.

Text S3. Summarised results from the study of multiple α-to-βratios.

1. Standard CARNIVAL

While the original study by Melas *et al.* chose parameters in a way that one fitted measurement justifies up to five additional nodes (α/β = 5), it is unclear how strongly changes in the node

penalty β affect the solutions obtained and why this particular ratio was selected. To evaluate the sensitivity of the method to changes in this penalty ratio, the mismatch penalty was set to $\alpha = 5$ and the effect of changes in node penalty tested around the parameter values of $\beta = [0; 2]$.

As expected by its definition, it was found that the number of nodes and interactions dropped with increasing node penalty (Figure ST1). In contrast, the overall interaction to node ratio and the number of models did not show a clear trend (results not shown). Only 45% of the perturbations (9 out of 20) solutions were derived from CPLEX with a node penalty of $\beta = 2$. Comparison between the solutions derived with each node penalty in each perturbation revealed that the solutions changed with modified node penalties. However, for the standard CARNIVAL benchmarking results, similar node penalties lead to similar results and the sign is not contradicting over all considered node penalties for 96.9% (3,955/4,082) of the nodes. This indicates that the node penalty has an effect which might result in alternative, but rarely contradicting solutions.

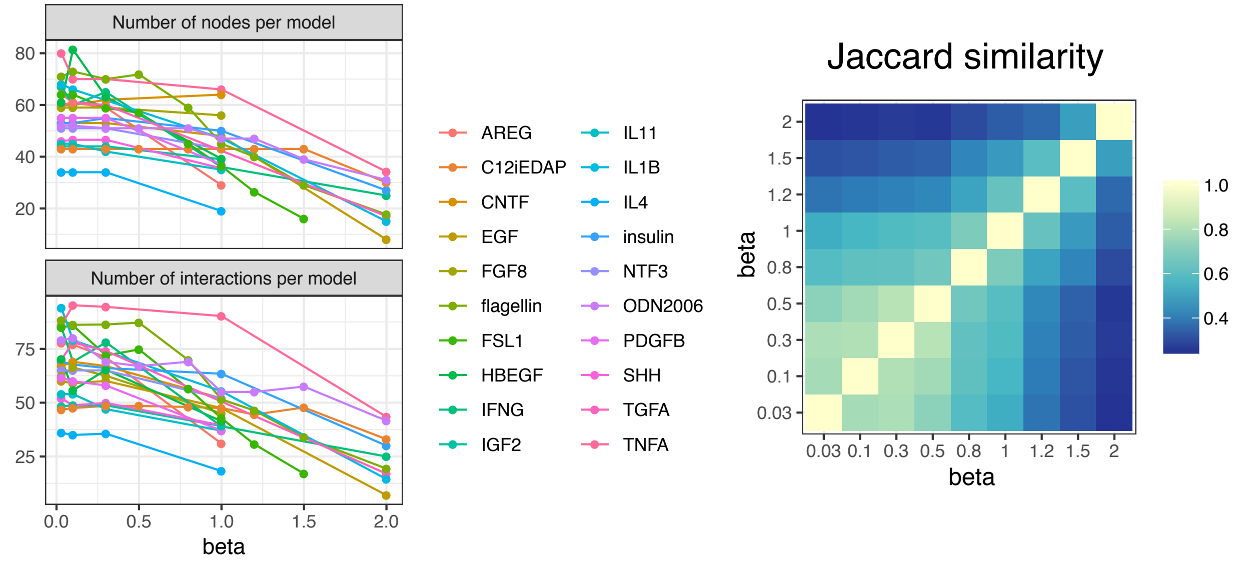

Figure ST1: Effects of changes in node penalty β. Overall, the number of nodes and interactions decreases with increasing node penalty, while the number of models and the interaction to node ratio do not show consistent trends. The Jaccard Index between the union of signed nodes obtained with different β is generally high between similar node penalties and decreases with increasing changes in β. Only the solutions with TF weights are compared in the figures, but similar trends were observed without TF weights.

To evaluate the effect of modifications on the node penalty β with respect to the ability of CARNIVAL to capture upstream alterations, the inferred node activities were first compared with the experimental phosphoprotein levels. However, only 3-4 of these phosphoproteins were predicted as dysregulated by CARNIVAL on average and could be matched with an activity inferred from experimentally measured phosphoprotein levels. Due to the limited availability of phosphoprotein levels, meaningful statistical testing could not be performed. As an alternative approach, the performance was evaluated based on the agreement with expected KEGG pathways with a two-step inference approach instead (see Methods).

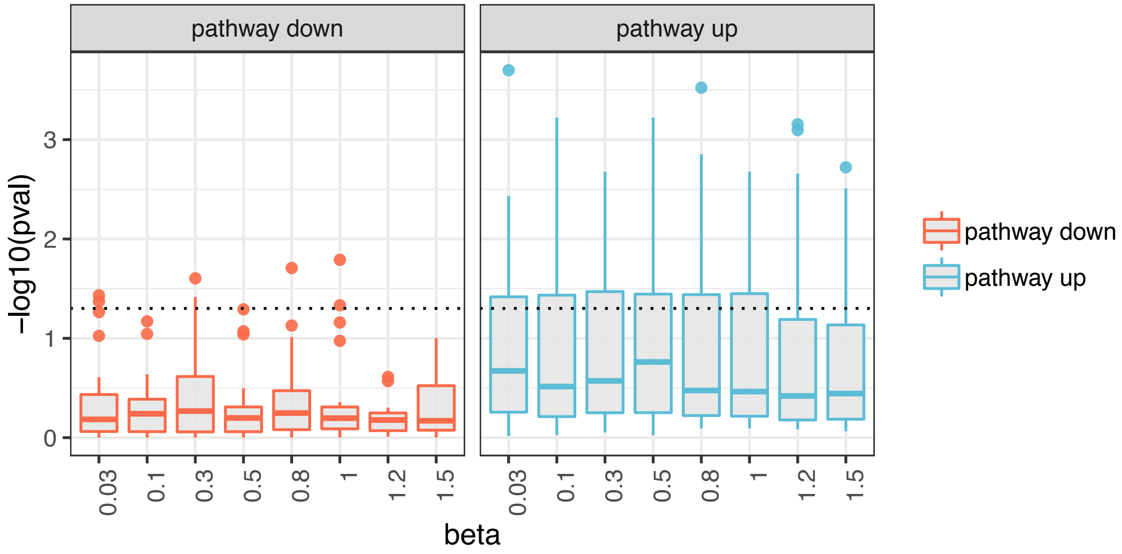

Figure ST2: Enrichment of the perturbation-attributed pathway set in dysregulated pathways inferred from CARNIVAL over different node penalty β. The KEGG enrichment of the attributed pathway set in the deregulated pathways shows overall similar results over different node penalty values. Generally, the attributed pathway set is slightly more enriched in activated than in inhibited pathways according to average p-values from 20 perturbations. The figure compares only solutions with TF weights, but similar trends were observed without TF weights.

Only minor fluctuations are observed in the enrichment of the perturbation-attributed pathway set over different node penalties and a general trend or optimum is not noticeable. Additionally, it should be noted that the perturbation-attributed pathway set averaging all p-values from 20 perturbations is slightly more significantly enriched in activated than inhibited pathways (Figure ST2), which means that the expected perturbation-attributed pathways are more enriched in activated pathways. For CARNIVAL with known perturbation, results with a node penalty of β = 1 are selected for display given that no improvement can be observed by deviating from the original mismatch to node penalty ratio of Melas *et al.*

In addition, we also explored the effect of different node penalties on the results from the two-step enrichment analyses. Results are shown in Supplementary Figure ST3 below.

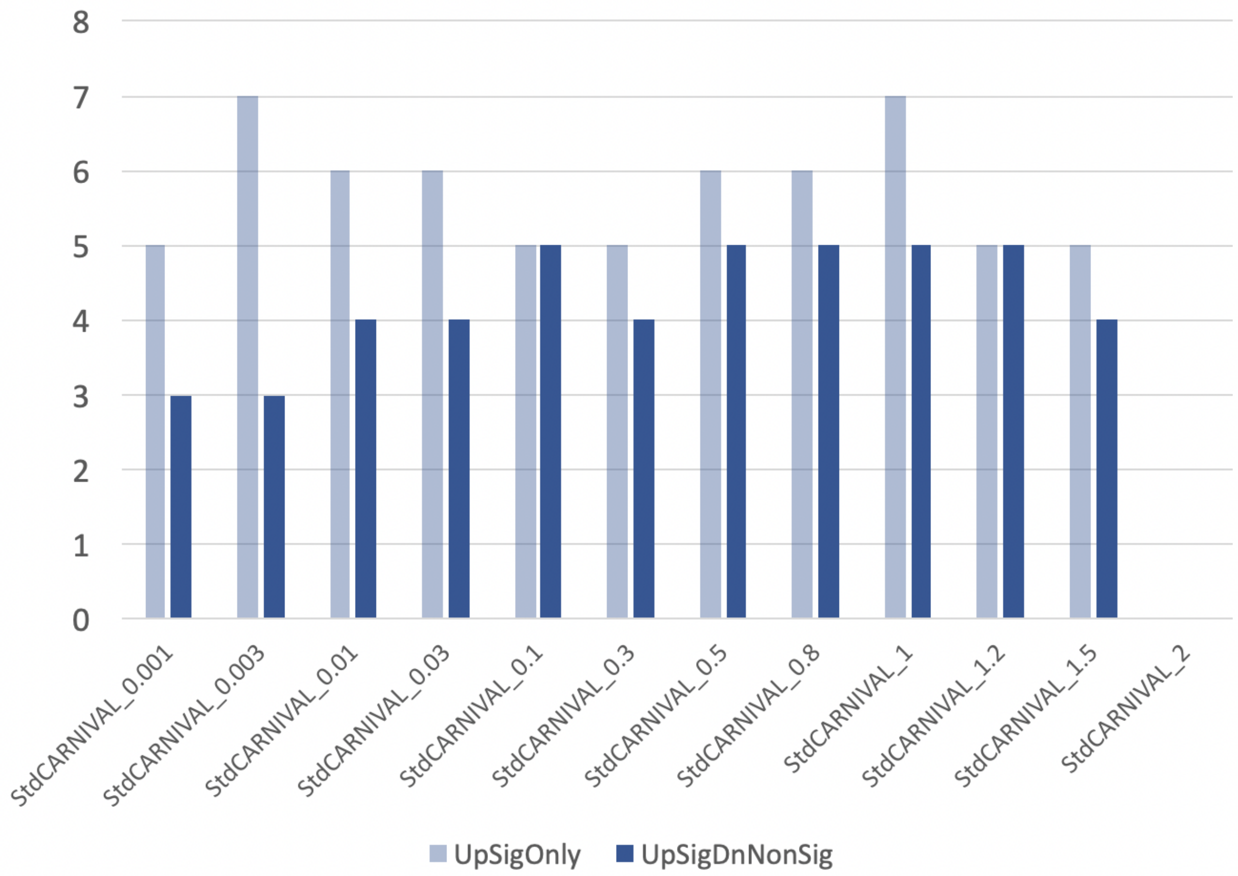

Figure ST3. Number of positive results in the SBVimprover benchmark study from the StdCARNIVAL pipeline with different node penalty 'beta' parameters. The results are classified according to the number of positive results with only significant enrichment of the up-regulated nodes in CARNIVAL network (UpSigOnly) and those with also insignificant enrichment of the down-regulated nodes in CARNIVAL network (UpSigDnNonSig).

According to the results in Figure ST3, we found slight deviations on the number of positive enrichment results among the range of betas from 0.001 to 1.5. Of note, there is no positive enrichment results from the StdCARNIVAL networks with beta=2. This is due to the much smaller size of these networks as demonstrated in Figure ST1 where there are insufficient numbers of nodes to perform the over-representation and enrichment analyses.

2. Inverse CARNIVAL

As a starting point, inverse CARNIVAL was first run with the previously used TF weights and a node penalty of $\beta = 1$. However, this resulted in networks with zero similarity to the previous solutions with known targets of perturbation in all cases. Further investigations revealed that all of the derived nodes are TFs inferred from DoRothEA and at the same time in the list of potential

input nodes (Figure ST3). Given that these nodes only possess outgoing but not incoming reactions they can only appear as input nodes, which explains why zero similarity between inverse and standard CARNIVAL was observed.

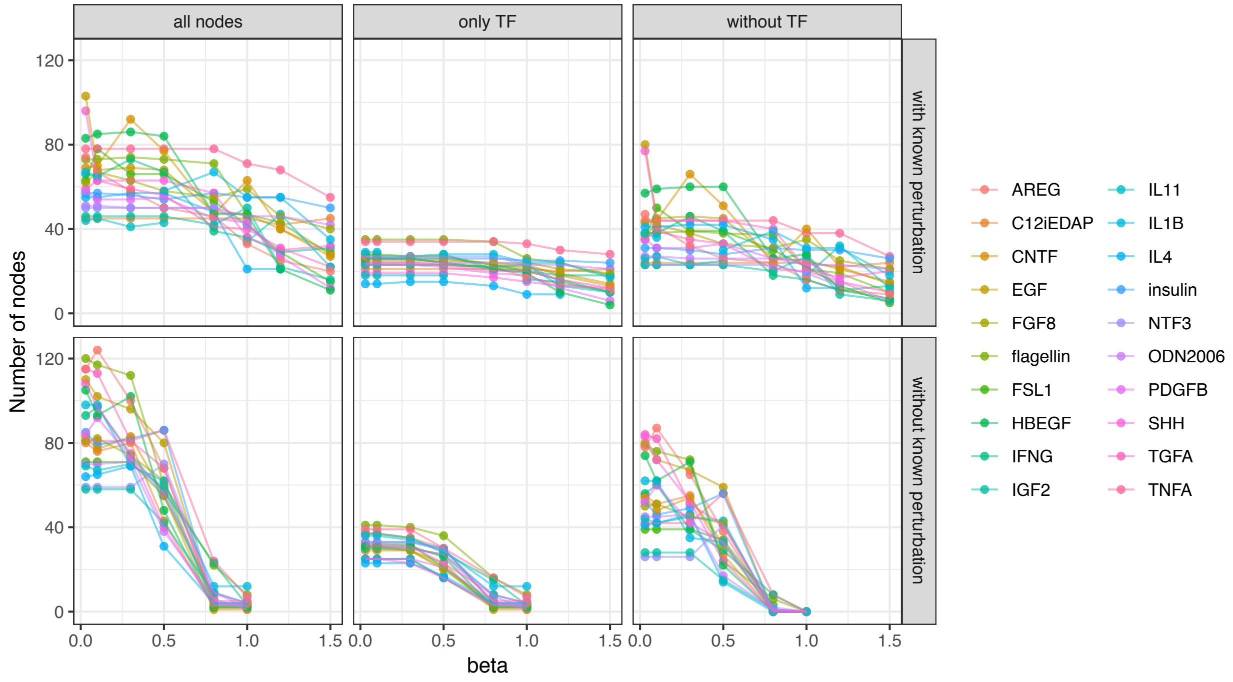

Figure ST4: Effects of changes in node penalty β on solution nodes. Without known perturbation, the inverse CARNIVAL pipeline is more sensitive towards changes in β. With a node penalty of 1, all appearing nodes are TFs predicted by DoRothEA, which are by definition not penalized.

In Figure ST4, the effect of the node penalty was also screened for inverse CARNIVAL and showed that the number of nodes dropped more rapidly with increasing node penalty. Also its solutions were hence more sensitive to adaptations in β than the ones of CARNIVAL with known perturbation. Given that clear trends or optima were again not identifiable by a comparison of KEGG enrichment performance over β (results not shown), a node penalty of $\beta = 0.5$ was chosen for further benchmarking given that this resulted in similar numbers of nodes compared to CARNIVAL with known perturbation.

Text S4. Benchmark CARNIVAL versus DoRothEA and PROGENy.

Given that the CARNIVAL pipeline relies on the processed information from DoRothEA and PROGENy, we therefore performed two independent studies to illustrate the performance of CARNIVAL in comparison to the ones of its constituting tools.

On the comparison of CARNIVAL versus DoRothEA, as DoRothEA also operates at the individual node/protein level (i.e. transcription factors [TFs]), we ran the two-step enrichment

analyses using CARNIVAL nodes (DoRothEA+PROGENy) versus using the original 50 input TFs from DoRothEA (Figure ST5).

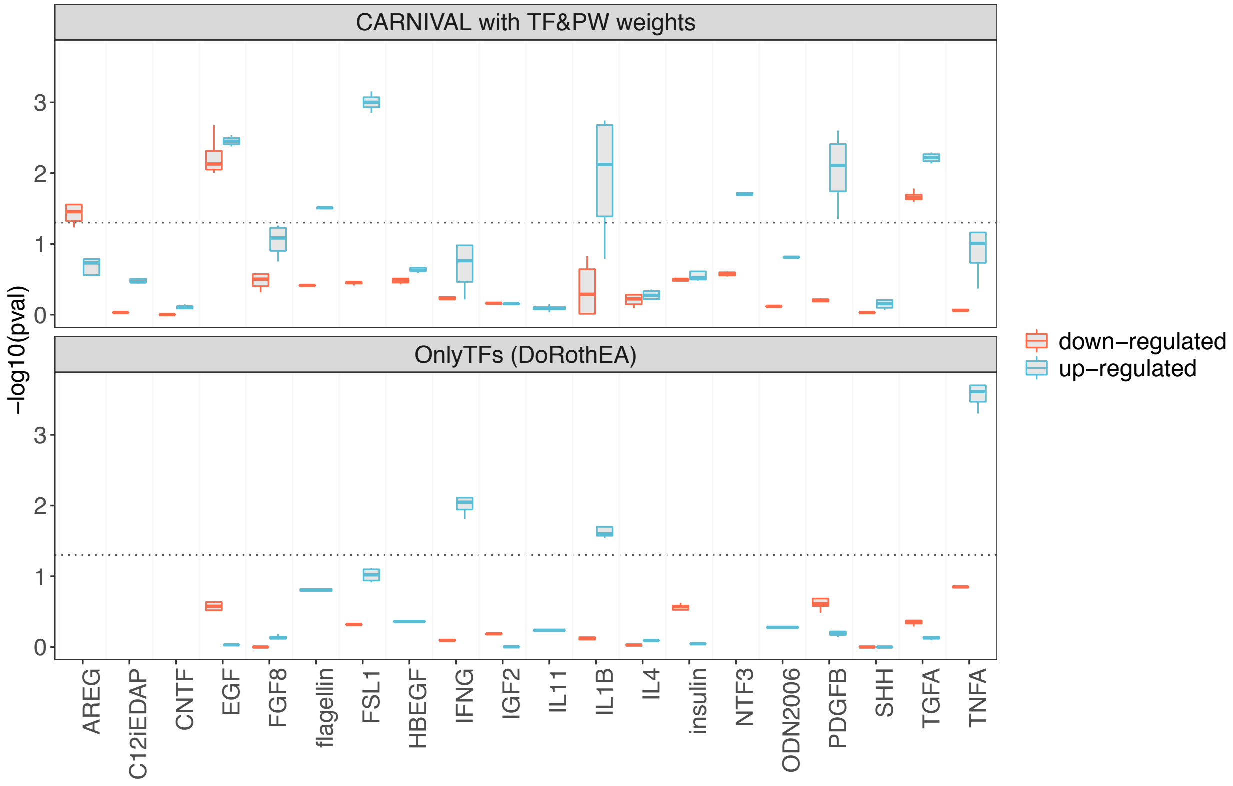

Figure ST5. Comparison of two-step enrichment results from StdCARNIVAL versus DoRothEA (OnlyTF).

Based on the results in Figure ST5, they showed that there is no enrichment from many stimuli/perturbations of the “OnlyTF” set: four TF have no results [AREG, C12iEDAP, CNTF, NTF3] while another four have only results from up-regulated TFs [flagellin, HBEGF, IL11, ODN2006]. Upon counting positive results (i.e. significant enrichment in the up-regulated part), stdCARNIVAL captured 5-7 enriched pathways while “onlyTF” captured only 3. Nevertheless, it should be noted that the “OnlyTF” results captured two pathways which CARNIVAL could not (IFNg and TNFa) and this is in line with the GSEA results (see Suppl. Figure S3). This reflects that certain information might be lost along the analytical pipeline moving from TFs towards pathway level - although CARNIVAL still delivered positive results for more cases.

Regarding the comparison of results from CARNIVAL versus pure pathway activity inference from PROGENy, we would like to highlight the fact that they are not directly comparable: CARNIVAL gives results at the individual node/protein level while PROGENy summarizes information and reports results at the pathway level. One indirect comparison could be performed by examining the (significant) pathway scores obtained by PROGENy and the p-values from over-representation analysis of CARNIVAL nodes of those pathways. We found four KEGG pathways in the curated MSigDB dataset that could represent PROGENy pathways: EGF (ErBB), MAPK (MAPK), PI3K (phosphoinositide) and Jak-Stat (JAK_STAT), see Table ST1.

Table ST1. Comparison of PROGENy scores versus the p-values (pVal) from over-representation analysis (ORA) of up-regulated (Up) and down-regulated (Dn) nodes based on StdCARNIVAL results in the expected KEGG pathways according to each perturbation.

Perturbation	Expected Pathways	PROGENy score	ORA-Up-pVal	ORA-Dn-pVal
AREG	EGFR	0.287	0.0546	0.1829
	MAPK	0.5588	0.1571	0.0523
	PI3K	0.7138	0.0908	NA
EGF	EGFR	1	0.0560	0.0697
	MAPK	1	0.0298	0.7037
	PI3K	-0.1734	0.1165	NA
IFNG	JAK.STAT	0.8826	0.0807	0.2852
IGF2	PI3K	-0.9272	0.1478	0.8854
IL4	JAK.STAT	0.9006	0.0139	0.5497
IL11	JAK.STAT	-0.925	0.0049	0.5676
	MAPK	0.7932	0.0004	0.1369
insulin	PI3K	1	0.2038	NA
TGFa	EGFR	1	0.0048	0.4137
	MAPK	1	0.0012	0.5916
	PI3K	1	0.4468	0.3208

In general, PROGENy scores make sense for most of the stimuli/perturbations in the SBVimprover dataset, with some exceptions. IL11 is known to activate JAK-STAT3 and subsequent ERK-MAPK pathways. The PROGENy score of JAK.STAT is negative for IL11 stimulation, while CARNIVAL captured correctly JAK.STAT including the downstream MAPK pathway. The case of IGF2 is less conclusive as IGF2 could bind both to IGF1R (activating PI3K) and IGF2R (leading to lysosomal degradation). PROGENy predicted that PI3K is strongly down-regulated but CARNIVAL predicted the opposite although not significantly. Lastly, EGF is known to activate PI3K but the PROGENy score was negative, while CARNIVAL showed a low (yet not significant) p-value on the up-regulated part. These cases represent the findings from PROGENy that might

be contradicting with known biology while CARNIVAL capture them better. In some cases though, PROGENy captured expected signals that were not strong enough in CARNIVAL (e.g. insignificant enrichment for PI3K pathway in the TGFa case).

In summary, These two studies showed that CARNIVAL, with the addition of network knowledge and subsequent computations, offers complementary results to the TF and pathway scores from DoRothEA and PROGENy, respectively. Nine out of 15 results from CARNIVAL enrichment agreed with the sign of PROGENy scores (p-value threshold at 0.1) while CARNIVAL identified the same positive enrichment of IL1B case versus DoRothEA. Nevertheless, it should be noted that the positive results from these two tools did not always add up to the ones from CARNIVAL. Therefore, we propose CARNIVAL users to perform functional analyses at different levels and analyze these results altogether to get a broader overview for further interpretation and validation.

Text S5. Fluorescence immunohistology.

For fluorescence immunohistology we used 3 biopsies collected from healthy renal transplantation donors and compared with 3 biopsies collected for primary diagnosis from IgAN patients. Patients had active IgAN to be included as demonstrated by IgA deposition in glomeruli. Renal needle biopsy specimens were initially collected in PBS to remove blood contamination upon biopsy. Washed tissue specimens were then fixed in normal formalin (48h) and subsequently embedded in paraffin blocks and sectioned on a microtome at a thickness of 10um. Sections were then deparaffinized and antigen-unmasked using a heat-mediated antigen retrieval buffer (Abcam) for 2h at 100^0C. Sections were washed 3 times, blocked in 10% donkey serum in PBS (1h) and incubated with appropriate primary antibodies (mouse anti-RhoA; and rabbit anti beta-catenin; both from Abcam. Goat anti-IgA1 from Sigma). Primary antibodies were used at 1:100 dilution in 10% donkey serum and incubated with sections for 16h. Sections were washed 3 times in PBS-tween and incubated for 2h using donkey anti-mouse (AlexaFluor 647), donkey anti-rabbit (AlexaFluor 568) and donkey anti-goat (AlexaFluor 488) IgGs. Finally, sections were washed 3 times and mounted using VectaShield mounting solution (H1000). Images were taken on a Zeiss Axioscope coupled to a TissueGnostics imaging suite and a 20x lens. The same channel settings were used for all imaged sections (100ms exposure for 488 and 400ms exposure for 568 and 647).

Text S6. Application of CARNIVAL to the CCLE dataset.

Recently, Ghandi et al. published an updated dataset on the Cancer Cell Line Encyclopedia (CCLE) which contains RNAseq data at the transcriptomics level as well as reverse phase protein array (RPPA) data at the (phospho-)proteomic level[7]. To illustrate that CARNIVAL is also applicable to RNAseq data in addition to the microarray datasets as demonstrated in the SBVimprover and IgAN studies, we applied CARNIVAL to contextualize regulatory signaling networks of 1019 cancer cell lines based on their basal gene expression. Subsequently, we compared average CARNIVAL nodes' activity to the level of phosphorylated proteins from RPPA experiment where we identified good correlations for many signaling proteins.

Regarding transcriptomics data processing, raw RNAseq count data were formatted into the digital gene expression (DGE) class with the function *DGElist* and the library size was generated with the function *calcNormFactors* from the *edgeR* package[8]. Then, the *voom* function from the *limma* package was applied to transformed count data into log2 count per million (logCPM) scaled by the library size[9]. We subsequently applied a z-score transformation of each gene across all cell lines in order to make the basal gene expressions of all cancer cell lines relatively comparable. The processed z-score transformed logCPM RNAseq data are compatible with the DoRothEA and PROGENy pipelines which allows the calculating of transcription factor and pathways scores, respectively, as the inputs for CARNIVAL.

As the CCLE dataset contains basal gene expressions of cancer cell lines, there are not clear targets of perturbation, and we thus applied the InvCARNIVAL pipeline using Omnipath as the prior knowledge network (PKN)[10]. Then, we correlated the average CARNIVAL nodes' activities to the level of phosphorylated proteins per antibody using Pearson and Spearman correlation measures. Complete results of correlation analyses are shown in Table ST2 and the plots of CARNIVAL node activities versus the level of phosphorylated proteins for top 2 correlated and anti-correlated antibodies are shown in Figure ST5.

Table ST2. Correlation measures between average CARNIVAL nodes' activities and their corresponding phosphoprotein levels from the RPPA experiment in the CCLE dataset. The name of targeted molecules which have more than one detecting antibodies were labelled by the tag numbers. The targeted molecules with corresponding antibodies were ranked based on their averaged Pearson and Spearman correlations in the Mean column.

Target	Antibody Name	Pearson	Spearman	Mean
ERBB2	HER2_pY1248_Caution	0.5464	0.6837	0.61505
EGFR_1	EGFR_pY1068_Caution	0.4214	0.4683	0.44485
YAP1	YAP_pS127_Caution	0.3631	0.3332	0.34815
JUN	c.Jun_pS73	0.3001	0.2885	0.2943
RPS6KA1_2	p90RSK_pT573_Caution	0.265	0.2831	0.27405
SHC1	Shc_pY317	0.34	0.1518	0.2459
STAT3	STAT3_pY705	0.1989	0.2905	0.2447
NFKB1	NF.kB.p65_pS536_Caution	0.2308	0.2524	0.2416
CHEK1	Chk1_pS345_Caution	0.2567	0.217	0.23685
ARAF	A.Raf_pS299_Caution	0.2854	0.177	0.2312

MDM2	MDM2_pS166	0.2328	0.1812	0.207
MTOR	mTOR_pS2448_Caution	0.1064	0.3	0.2032
AKT1_3	PRAS40_pT246	0.159	0.1865	0.17275
AKT2_1	Akt_pS473	0.1527	0.1366	0.14465
RAF1	C.Raf_pS338	0.1446	0.1353	0.13995
SRC_1	Src_pY416_Caution	0.1463	0.1289	0.1376
MAPK1_2	p38_pT180_Y182	0.1144	0.118	0.1162
MAPK8	JNK_pT183_Y185	0.1131	0.1162	0.11465
FOXO3	FOXO3a_pS318_S321_Caution	0.0943	0.1174	0.10585
MAPK1_1	MAPK_pT202_Y204	0.1097	0.0959	0.1028
PEA15	PEA15_pS116	-0.0465	0.2476	0.10055
RPS6KA1_1	p90RSK_pT359_S363_Caution	0.0862	0.1001	0.09315
MAPK14	p38_pT180_Y182	0.0981	0.0844	0.09125
AKT1_1	Akt_pS473	0.1009	0.0806	0.09075
AKT1_2	Akt_pT308	0.095	0.0752	0.0851
MAPK3	MAPK_pT202_Y204	0.0626	0.0887	0.07565
GSK3B_2	GSK3_pS9	0.0622	0.0825	0.07235
CTNNB1	beta.Catenin_pT41_S45	0.0509	0.0669	0.0589
AKT3_1	Akt_pS473	0.0348	0.0828	0.0588
EGFR_2	EGFR_pY1173	0.0603	-0.0101	0.0251
GSK3B_1	GSK3.alpha.beta_pS21_S9	-0.0331	-0.0052	-0.01915
GSK3A_1	GSK3.alpha.beta_pS21_S9	-0.0296	-0.0359	-0.03275
GSK3A_2	GSK3_pS9	-0.0425	-0.0352	-0.03885
AKT2_2	Akt_pT308	0.0183	-0.0968	-0.03925

PRKAA1	AMPK_pT172	-0.0514	-0.0657	-0.05855
CHEK2	Chk2_pT68_Caution	-0.0834	-0.0997	-0.09155
SRC_2	Src_pY527	-0.1092	-0.0818	-0.0955
RPS6KB1	p70S6K_pT389	-0.2219	-0.0768	-0.14935
PRKCA	PKC.alpha_pS657_Caution	-0.173	-0.1565	-0.16475
AKT3_2	Akt_pT308	-0.1356	-0.2073	-0.17145
ESR1	ER.alpha_pS118	-0.2279	-0.2962	-0.26205
PRKCD	PKC.delta_pS664	-0.2975	-0.2771	-0.2873
BRAF	B.Raf_pS445	-0.4018	-0.3669	-0.38435
RB1	Rb_pS807_S811	-0.4991	-0.52	-0.50955
PTPN11	SHP.2_pY542_Caution	-0.9368	-0.4	-0.6684

Given that the average CARNIVAL nodes' activities which were generated from the family of alternative solutions might be sensitive to noise, we therefore selected only the corresponding phosphorylated protein levels of the CARNIVAL nodes which have consistent activities of either 1 or -1 to ensure that all network solutions agree whether the respective nodes are up- or down-regulated, respectively. We then performed an additional statistical analysis with the Student's t-test (using the function *t.test* from the built-in *stats* R-package) with adjusted p-value with FDR to determine if there are any significant in the phosphorylated protein levels between the 1 and -1 groups. Subsequently, we plotted the effect size (t-value) and the minus log10 p-value of the results as shown in a volcano plot in Figure ST6.

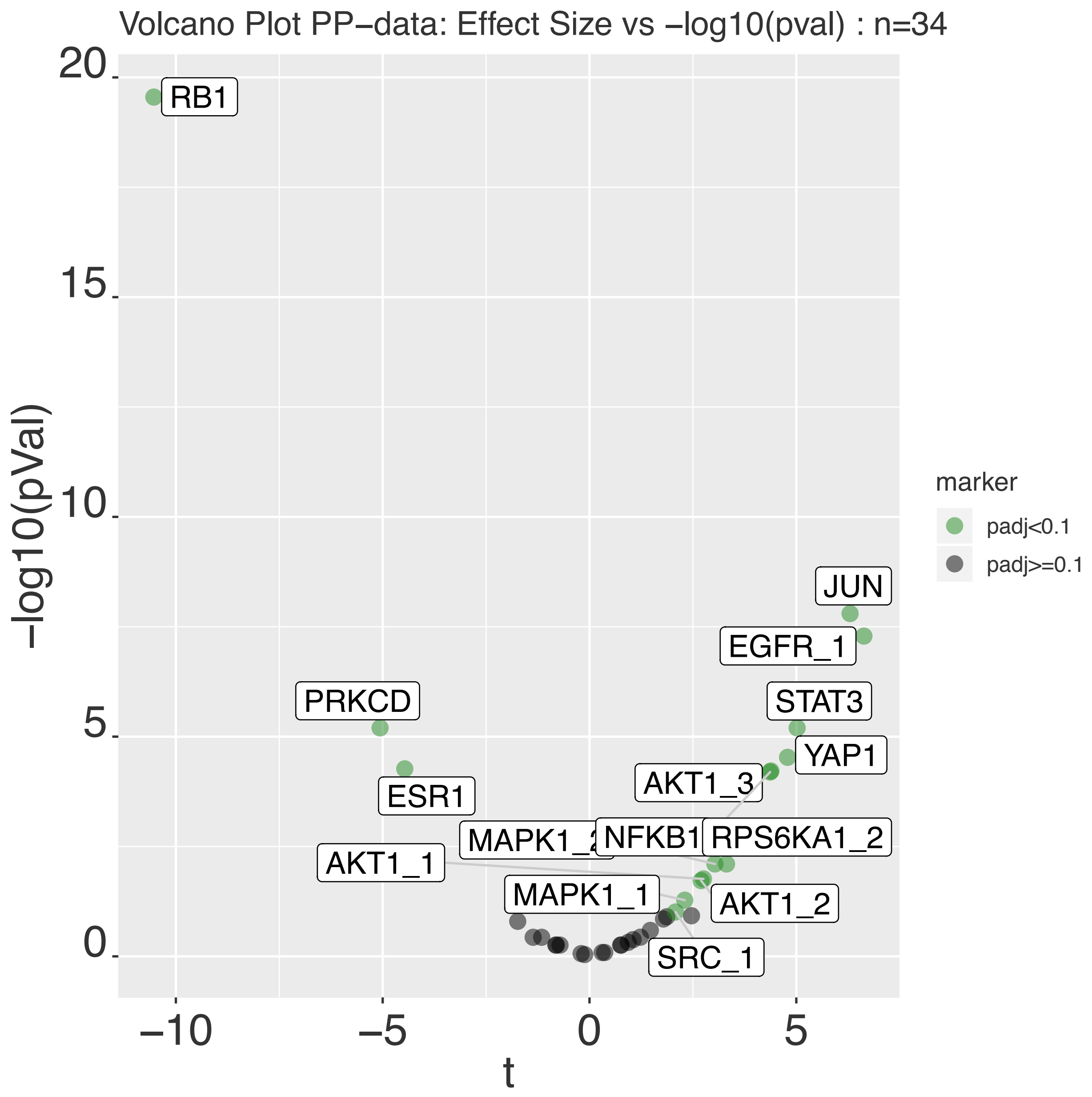

Figure ST6. Effect size and significance levels based on the differences in phosphoprotein levels of the corresponding CARNIVAL nodes with average activities of 1 and -1. A volcano plot comparing between the effect size (t-values) and the significance level (p-values) in minus log10 scale was demonstrated. The signaling proteins with p-value less than 0.1 were labeled in green and highlighted with name tags.

According to the results in Figure ST6, the majority of signaling proteins do not pass the significant level of FDR=0.1. For those which passed this significance threshold, 12 molecules have positive t-values i.e. the phosphorylated levels of the node/molecule which have CARNIVAL node activity of 1 are significantly higher than those of the nodes with CARNIVAL nodes' activity being -1. On the other hand, we also observed 3 molecules which have significant negative t-values i.e. RB1, PRKCD and ESR1. A possible explanation for the RB1 case lies on the fact regarding the state of

phosphorylation versus the activity of this signaling protein. RB1 binds to E2F transcription factors which control cell cycle in a hypophosphorylated state. Upon the stimulation of ligands that promote cell division, RB1 is hyperphosphorylated at the pS807 and pS811 phosphosites which will then release E2Fs to initiate the cell cycle circuit[11]. The phosphorylated state of RB1 is therefore inverse to its state of activity that CARNIVAL predicts. The observed inverse correlation of this RB1 is hence explainable. For PRKCD, the role of the detected phosphosite (S664) was not well-documented whether it is activatory or inhibitory. The function of measured phosphosite for ESR1 (S118) was annotated as activatory but the activity of this node was indeed defined by DoRothEA as ESR1 is a transcription factor. Thus, in this case study CARNIVAL correctly captured the activation of 87 % (13 out of 15) proteins (including RB1). In particular, the components of major signaling pathways including ERBB, PI3K/Akt, Jak/Stat, NFkB and cell cycle were still correctly captured.

To conclude, this independent CARNIVAL study demonstrated that the CARNIVAL pipeline is also capable of integrating RNAseq datasets as inputs. With proper data scaling, CARNIVAL can also be applied to generate contextualized regulatory signaling networks from single-sample gene expression data such as the updated CCLE dataset. In addition, we showed that CARNIVAL nodes' activities correlate well to the corresponding phosphorylated level of the majority of signaling proteins. These findings highlight the flexibility of CARNIVAL for data integration with diverse data types and also ensure that the CARNIVAL nodes' activities correspond to the actual activity of signaling proteins that they aim to represent.

References

1. Edgar, R., Domrachev, M. & Lash, A. E. Gene Expression Omnibus: NCBI gene expression and hybridization array data repository. *Nucleic Acids Res.* **30**, 207–210 (2002).
2. Berthier, C. C. *et al.* Cross-species transcriptional network analysis defines shared inflammatory responses in murine and human lupus nephritis. *J. Immunol.* **189**, 988–1001 (2012).
3. Hodgin, J. B. *et al.* The molecular phenotype of endocapillary proliferation: novel therapeutic targets for IgA nephropathy. *PLoS One* **9**, e103413 (2014).
4. Liu, P. *et al.* Transcriptomic and Proteomic Profiling Provides Insight into Mesangial Cell Function in IgA Nephropathy. *J. Am. Soc. Nephrol.* **28**, 2961–2972 (2017).
5. Woroniecka, K. I. *et al.* Transcriptome Analysis of Human Diabetic Kidney Disease. *Diabetes* **60**, 2354–2369 (2011).
6. Melas, I. N. *et al.* Identification of drug-specific pathways based on gene expression data: application to drug induced lung injury. *Integr. Biol.* **7**, 904–920 (2015).
7. Ghandi, M. *et al.* Next-generation characterization of the Cancer Cell Line Encyclopedia. *Nature* **569**, 503–508 (2019).
8. Robinson, M. D., McCarthy, D. J. & Smyth, G. K. edgeR: a Bioconductor package for differential expression analysis of digital gene expression data. *Bioinformatics* **26**, 139–140 (2010).
9. Ritchie, M. E. *et al.* limma powers differential expression analyses for RNA-sequencing and microarray studies. *Nucleic Acids Res.* **43**, e47 (2015).
10. Türei, D., Korcsmáros, T. & Saez-Rodriguez, J. OmniPath: guidelines and gateway for literature-curated signaling pathway resources. *Nat. Methods* **13**, 966–967 (2016).
11. Chinnam, M. & Goodrich, D. W. RB1, Development, and Cancer. *Current Topics in Developmental Biology* 129–169 (2011). doi:10.1016/b978-0-12-380916-2.00005-x
